# Valorization of Agri-Food Waste from Pigment-Rich Root Vegetable in Integrated EU Biorefinery Systems

**DOI:** 10.3390/foods15081432

**Published:** 2026-04-20

**Authors:** Ecaterina Matei, Loredana Cosma, Maria Râpă, Anda-Sorina Calotă, Andra Mihaela Predescu, Alecsia Stoica, George Coman

**Affiliations:** 1 Faculty of Materials Science and Engineering, National University of Science and Technology Politehnica Bucharest, 060042 Bucharest, Romania; ecaterina.matei@upb.ro (E.M.); maria.rapa@upb.ro (M.R.); andra.predescu@upb.ro (A.M.P.); george.coman@upb.ro (G.C.); 2 Biotechnical Systems Engineering Doctoral School, National University of Science and Technology Politehnica Bucharest, 313 Splaiul Independentei, 060042 Bucharest, Romania; anda_sorina.calota@stud.sim.upb.ro

**Keywords:** agri-food waste, root vegetables, pigments, anthocyanins, betalains, circular biorefinery

## Abstract

Agri-food processing in Europe generates large quantities of organic residues that remain insufficiently valorized despite their significant biochemical potential. Among these, wastes derived from root vegetables and anthocyanin-rich crops represent a distinct category of non-lignocellulosic biomass characterized by high moisture content, low lignin levels, and substantial concentrations of fermentable carbohydrates and bioactive compounds. This review provides a systematic overview of the origin, composition, and valorization potential of these residues, as well as extraction methods, with particular emphasis on root vegetable processing wastes and pigment-rich agri-food by-products. Valorization options are discussed within an integrated biorefinery perspective, particularly for specific compositional characteristics of the investigated waste streams related to suitable recovery strategies, followed by the conversion of post-extraction residues into secondary products and bioenergy. These options are evaluated in relation to the origin, biochemical profile, and valorization potential of each waste stream, as detailed in the dedicated sections of the review. Cascading utilization strategies are highlighted as a means to improve resource efficiency and reduce environmental burdens compared to single-route treatment options. By integrating information on feedstock characteristics and processing pathways, this review contributes to a better understanding of non-lignocellulosic agri-food wastes and supports the development of sustainable valorization strategies in the European circular bioeconomy.

## 1. Introduction

Agri-food systems generate large quantities of organic residues that remain underutilized despite their significant biochemical and energetic potential. Wastes from root vegetables and anthocyanin-rich crops represent heterogeneous non-wood biomass streams that are still predominantly directed toward low-value pathways such as composting or landfilling, despite their suitability for integrated biorefinery valorization due to high carbohydrate and bioactive content [1,2,3,4,5]. Agri-food waste generation represents a major environmental and economic challenge, with global food losses estimated to reach nearly USD 1 trillion annually, and approximately 88 million tonnes of food waste generated each year in the European Union [6,7,8,9]. Fruits and vegetables account for a substantial share of this waste, reflecting both high production volumes and significant losses along the supply chain. Across the European Union, approximately 58–59 million tonnes of food waste were generated annually during the 2023–2025 period, corresponding to roughly 130–132 kg per capita per year [10,11]. Vegetables contribute close to one-fifth of total food waste, with potatoes, roots, and tubers alone accounting for about 11%, thereby constituting a substantial yet underutilized non-lignocellulosic biomass stream [12]. This potential is reinforced by production data: total vegetable harvests exceeded 62 million tonnes in 2024, including significant volumes of root, tuber, and bulb crops such as carrots, onions, radishes, and garlic, alongside extensive sugar beet cultivation [13,14]. Together, these supply chains generate considerable quantities of biodegradable residues that remain insufficiently valorized despite their suitability for material recovery and bioenergy conversion.

While research on lignocellulosic biomass valorization remains extensive, attention to root-derived and pigmented vegetable residues is comparatively limited, even though such streams exhibit favorable properties—including high moisture, starch/sugar content, and low lignin—that make them amenable to biological conversion and cascading use [1,15]. Also, agro-industrial by-products are increasingly recognized as sustainable sources of high-value bioactive compounds within circular economy and upcycling frameworks [16,17,18,19,20].

In the European context, fruit and vegetable wastes are increasingly recognized for their potential to yield value-added molecules, natural pigments, and metabolites that support circular economy aims. Yet, most valorization efforts still treat compound extraction and energy recovery as separate domains, limiting opportunities for holistic, multi-product biorefinery strategies.

In Europe, a strong agricultural base in root vegetable cultivation with expanding processing capacity and limited implementation of advanced waste valorization technologies has been encountered [21]. Some analyses indicate that agricultural and food wastes remain predominantly managed through disposal or low-efficiency recovery routes, despite structural potential for higher-value utilization under circular economy frameworks [22,23,24]. For example, in Romania, a persistent gap in consumer information and market accessibility—together with the growing generation of organic agri-food waste—highlights the need for improved education, supply diversification, and integrated valorization strategies to support sustainable market expansion and circular resource use [25,26]. However, high-value waste management and advanced valorization pathways remain comparatively underdeveloped, resulting in continued reliance on low-efficiency recovery or disposal routes [13]. Collectively, the European trends highlight a clear opportunity to advance integrated and sustainable valorization strategies—particularly cascading biorefinery approaches capable of combining material extraction with energy generation from non-lignocellulosic root and pigmented vegetable residues—thereby supporting EU food waste reduction objectives and broader circular bioeconomy transition.

Focusing on non-lignocellulosic, root-derived, and pigmented wastes is justified by three key factors. First, these waste streams offer technical advantages, such as high biodegradability and compatibility with low-energy biological processes, which can reduce the need for intensive pretreatment [27,28,29,30]. Second, they support genuine cascading valorization by enabling recovery of high-value compounds (e.g., pigments and bioactive compounds) without precluding subsequent energy or fuel production [31,32,33,34]. Third, their utilization aligns with EU policy priorities on food waste reduction, renewable energy integration, greenhouse gas mitigation, and circular bioeconomy development, as reflected in sustainable waste management directives and SDG targets [33,35,36].

From a geographical perspective, this review is primarily framed within the European Union, where agri-food waste generation is characterized by high volumes of root vegetable- and fruit-derived residues within structured supply chains and regulated waste management systems. The findings are particularly relevant for temperate regions with similar agricultural profiles; however, caution is required when extrapolating to other contexts. For instance, in Northern European regions (e.g., Scandinavia), lower ambient temperatures may slow biological conversion processes such as anaerobic digestion or fermentation, increasing energy requirements for process heating and thereby influencing the overall carbon footprint [37,38]. In contrast, tropical regions (e.g., Southeast Asia, Sub-Saharan Africa, or parts of Latin America) are characterized by higher temperatures and faster biodegradation rates, but also by different biomass compositions (often more lignocellulosic) and less developed waste management infrastructures, which may limit the applicability of integrated biorefinery systems [39,40]. These regional differences have direct implications for greenhouse gas emissions, process efficiency, and life cycle environmental performance, highlighting the importance of geographically adapted valorization strategies under changing climate conditions.

While several recent studies have reviewed agri-food waste valorization [2,5,31], these contributions typically focus either on lignocellulosic biomass or on specific valorization pathways such as bioactive-compound extraction or bioenergy production.

The novelty of the present review lies in three main aspects: (i) its focus on non-lignocellulosic residues, particularly root vegetable and anthocyanin-rich wastes, which are comparatively underrepresented in the literature; (ii) the integration of these distinct waste streams within a unified cascading biorefinery framework; and (iii) the explicit contextualization within the European Union, highlighting regional opportunities and constraints.

These elements provide a system-level perspective that complements and extends the existing literature.

Accordingly, this review first focuses on the origin and composition of the selected agri-food waste streams, and then examines their potential valorization pathways within integrated systems. The review provides an integrated perspective on root vegetable and pigment-rich residues, a waste category rarely addressed in a unified framework.

## 2. Bibliometric Analysis and Research Trends

In this review paper, the literature data source was the Web of Science Core Collection database, and the keywords used were: vegetable waste, root vegetables, anthocyanins, and biorefinery.

**Figure 1:**
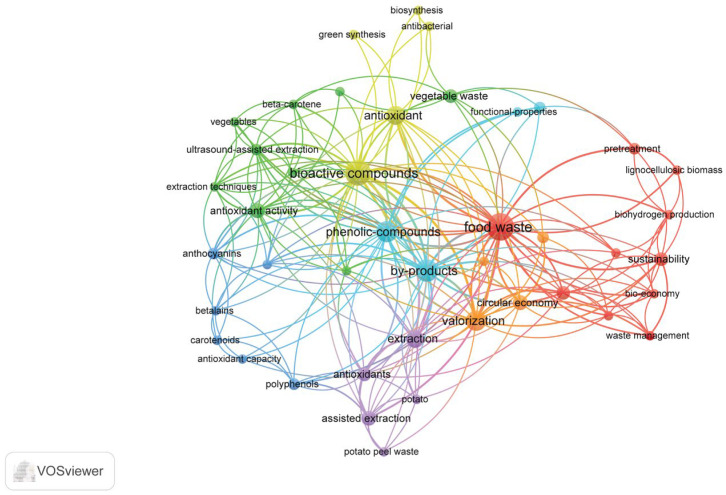
The relationships between the most frequently used keywords in the analyzed literature, generated with VOSviewer.

A total of 6949 document records were found from 2015 to 2026, from which 493 keywords were extracted by VOSviewer (version 1.6.20). The minimum number of occurrences was set to 2 to ensure that relevant concepts were retained in the visualization while excluding keywords that appeared only once and therefore had limited significance within the dataset. Thus, 103 keywords were identified as meeting this threshold. Based on their co-occurrence relationships, these keywords were organized into seven clusters, highlighting the main research themes present in the analyzed literature. The visualization of the keyword network and the identified clusters is presented in Figure 1.

The identified clusters reveal the main research directions in the field, including food waste management and sustainability, bioactive compounds and antioxidant properties, extraction and recovery techniques, and the valorization of agri-food by-products within the circular economy framework.

Cluster 1: Food waste (red color) is centered around the keyword food waste and includes terms such as sustainability, bioeconomy, waste management, biohydrogen production, lignocellulosic biomass, and pretreatment [23,27,29,30,36,41,42,43]. This cluster reflects research focused on the sustainable management and conversion of food waste into valuable resources. Cluster 2: Bioactive compounds and antioxidants (green color) includes keywords such as bioactive compounds, antioxidant activity, beta-carotene, vegetables, vegetable waste, and ultrasound-assisted extraction [6,18,31,33,43,44,45,46,47,48,49,50,51,52,53]. This cluster highlights research investigating bioactive molecules derived from plant-based food waste, particularly compounds with antioxidant properties. Cluster 3: Phenolic compounds (light blue color) includes terms such as by-products and functional properties [18,54,55,56,57,58,59,60,61,62]. This cluster represents research focusing on the chemical characterization and functional potential of phenolic compounds recovered from food processing residues. Cluster 4: Valorization and circular economy approaches (orange) includes keywords such as valorization and circular economy and emphasizes strategies aimed at transforming food waste and agro-industrial residues into value-added products [4,8,18,23,33,34,63,64]. Cluster 5: Extraction techniques (purple color) contains keywords such as extraction, assisted extraction, and potato peel waste and reflects extraction methods for recovering bioactive compounds from food waste materials [58,64,65,66,67,68,69,70]. Cluster 6: Natural pigments and antioxidant capacity (blue) includes terms such as anthocyanins, betalains, carotenoids, polyphenols, and antioxidant capacity [58,64,65,66,67,68,69,70]. This cluster focuses on natural pigments and phenolic compounds present in plant-based food waste, which are widely studied due to their nutritional and functional properties, as well as their potential applications as natural colorants and antioxidants in food products. Cluster 7: Green synthesis and bioactive applications (yellow color) contains keywords such as antioxidant, biosynthesis, green synthesis, and antibacterial [3,19,46,53,62,71,72]. This cluster highlights research exploring green and sustainable approaches for the synthesis and application of bioactive compounds derived from food waste.

Among all keywords, “food waste” appears as the most central node in the network, indicating its pivotal role in connecting multiple research themes. Its large node size reflects a high number of occurrences in the analyzed publications, while the numerous links with other keywords demonstrate strong co-occurrence relationships.

**Figure 2:**
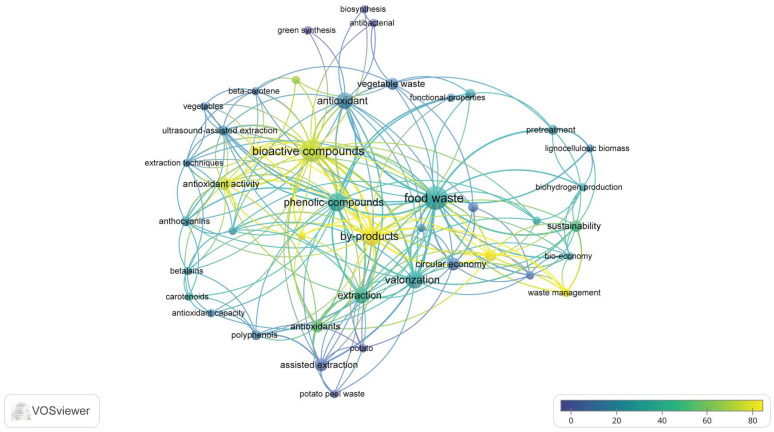
Overlay visualization of keyword co-occurrence related to root vegetable waste valorization and anthocyanin recovery, generated using VOSviewer.

The overlay visualization generated using VOSviewer illustrates the temporal evolution of research topics related to food waste valorization, as presented in Figure 2.

In this visualization, the color gradient represents the average publication year of the keywords, ranging from earlier topics (displayed in blue) to more recent research trends (displayed in yellow).

**Figure 3:**
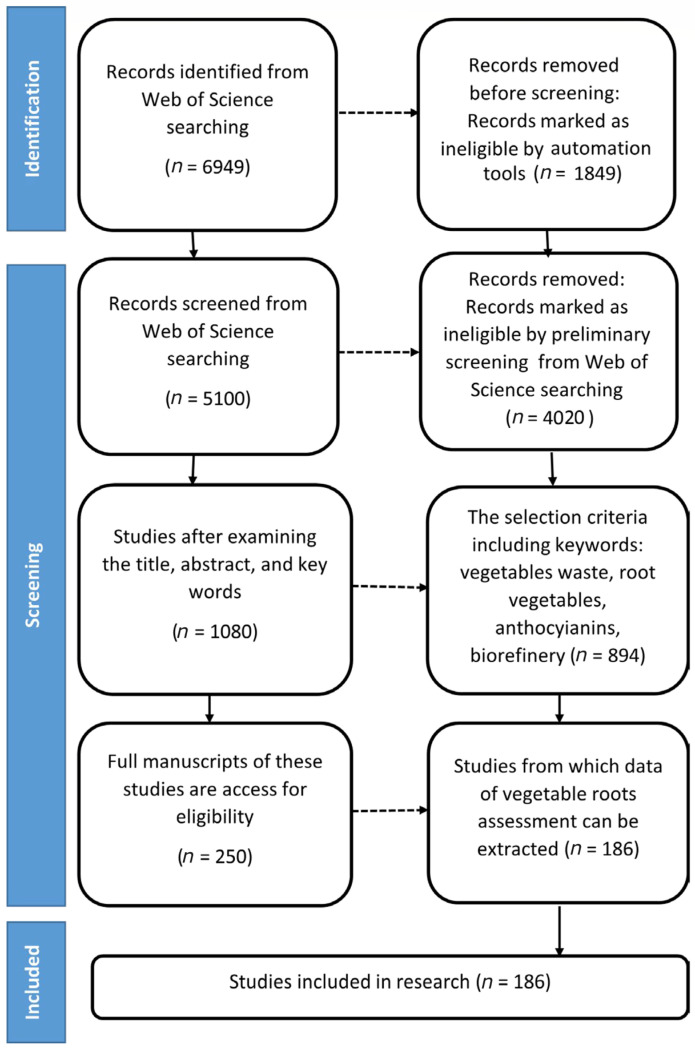
Studies included in the research from the Web of Science databases, in the period from 2015 up to the present, according to PRISMA.

The literature selection process was conducted in accordance with the PRISMA guidelines. In the initial stage, 6949 articles were identified from scientific databases. After removing duplicates (n = 1849), 5100 articles remained for the screening stage. Following an evaluation of the title and abstract, 4020 articles were excluded, and 1080 were selected for full-text analysis. Of these, 910 articles were eliminated based on eligibility criteria, resulting in a total of 186 studies included in the systematic review (Figure 3).

The results indicate that earlier studies in the field primarily focused on extraction techniques and specific bioactive compounds, including anthocyanins, carotenoids, polyphenols, and antioxidant capacity. These topics, shown in darker blue tones, reflect the initial scientific interest in identifying and characterizing valuable compounds derived from plant-based residues and vegetable waste.

More recent research trends, highlighted in yellow tones, are associated with concepts such as bioactive compounds, circular economy, and waste management. This indicates a growing scientific interest in integrating food waste valorization into sustainable resource management and circular bioeconomy frameworks, emphasizing the transformation of food waste into value-added products and sustainable materials.

This review is based on a dataset of 186 publications that reflect the selected search keywords and database filtering applied in Web of Science; the obtained results provide valuable insights into the main research themes and emerging trends related to food waste valorization and the recovery of bioactive compounds.

## 3. Waste Streams Considered: Origin, Composition and Valorization Potential

### 3.1. Root Vegetable Processing Wastes

Root vegetable processing produces considerable quantities of organic residues, mainly consisting of peels, trimmings, pulp, and starch-rich wash waters. Potato, beetroot, and carrot wastes are the most extensively investigated due to their abundance and comparatively uniform composition. These residues generally contain high moisture levels (approximately 70–85%) and substantial amounts of readily fermentable carbohydrates—primarily starch and soluble sugars—making them well suited for biological conversion pathways [73,74], as well as anthocyanin.

These wastes, such as peels and processing residues from potato, beetroot, carrot, and other tubers, are generally characterized by high moisture content, abundant starch, dietary fiber, essential micronutrients, and soluble sugars, and strong biodegradability. Likewise, residues from anthocyanin-rich vegetables, including purple carrot and purple potato, contain considerable concentrations of natural pigments and polyphenolic compounds with well-established antioxidant activity. Also, their relatively long refrigerated shelf life enables broad distribution and large-scale processing within agri-food supply chains, which in turn generates substantial quantities of residues during production, handling, and industrial processing, including peels, trimmings, and rejected roots [73]. These features enhance biodegradability and make root vegetable processing residues particularly suitable for biological conversion and integrated valorization within circular biorefinery systems [73].

#### 3.1.1. Production Scale and Availability

In Europe, root vegetable processing residues represent an important fraction of agri-food waste streams, particularly in regions with established potato and sugar beet processing industries. The industrial-scale processing associated with these crops results in the continuous generation of root vegetable processing wastes, making them consistently available feedstocks for valorization across different regions and seasons [75,76].

Root vegetables such as potato, carrot, sugar beet, parsnip, and beetroot are among the most widely cultivated and processed crops worldwide and play a central role in agri-food supply chains. Their large production volumes translate into substantial quantities of residues generated during industrial processing. Within the European Union, two major root crops dominate agricultural production: sugar beet, cultivated on approximately 1.6 million hectares in 2024, and potatoes, covering around 1.4 million hectares. In contrast, other root crops—including fodder beet, fodder kale, rutabaga, fodder carrot, and turnips—are considered niche or specialist crops, collectively occupying only about 0.1 million hectares [13]. The EU also holds a leading position in global sugar beet production, contributing roughly 50% of total worldwide beet sugar output [54,74,77,78,79].

**Figure 4:**
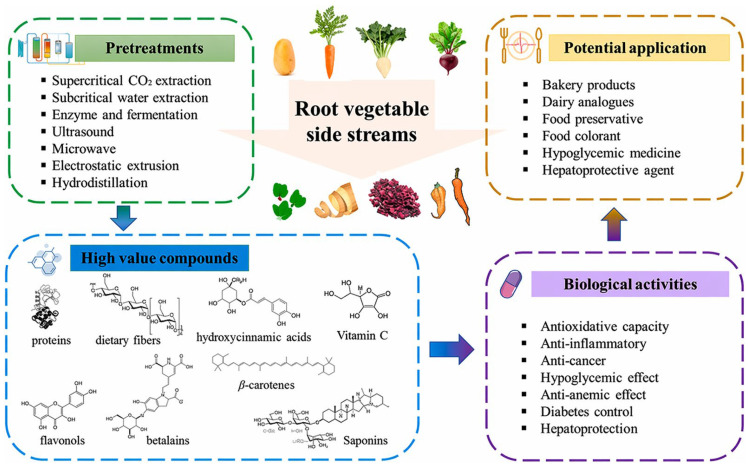
Pretreatments adapted in current studies on valorizing nutrients and bioactive compounds in root vegetables, and their biological activities and potential applications [80].

From a valorization perspective, root vegetable wastes can follow multiple pathways. Direct biological conversion routes, such as anaerobic digestion and dark fermentation, allow efficient energy recovery, while pre-extraction of bioactive compounds or starch can enhance overall system value. However, such cascading approaches require careful process integration to avoid excessive energy demand or loss of fermentability [34,76,80]. An example of pretreatment stages in order to valorize nutrients and bioactive compounds in root vegetables is illustrated in Figure 4.

Large production volumes translate into substantial quantities of processing residues generated along industrial value chains. Potato processing wastes, including peels and rejected tubers, are characterized by high starch content and low lignin levels, resulting in favorable biodegradability [77,81,82,83]. Beetroot and carrot residues present a more diverse composition, combining fermentable carbohydrates with structural polysaccharides and bioactive compounds. In addition, beetroot wastes are notable for their content of betalains and phenolic compounds, which may influence downstream biological processes depending on concentration and pretreatment strategy [44,45,55,78].

From a compositional perspective, root vegetable processing wastes are characterized by high moisture content (typically 70–85%) and a substantial proportion of readily fermentable carbohydrates, mainly starch and soluble sugars. Potato processing residues exhibit particularly high starch content and low lignin levels, resulting in favorable biodegradability and high conversion efficiency in biological processes [73,77,84].

Carrot and beetroot residues present a more heterogeneous matrix, combining fermentable carbohydrates with structural polysaccharides, proteins, and minor bioactive components [80,85,86]. These characteristics distinguish root vegetable residues from lignocellulosic biomass and contribute to their suitability for biological conversion and integrated valorization pathways [55,73].

Seasonal variability, cultivar differences, and processing technologies can significantly influence the composition and physicochemical properties of root vegetable processing wastes, leading to spatial and temporal heterogeneity across supply chains.

However, several of these streams—particularly leaves, pomace, and rejects—remain poorly characterized from a chemical and valorization perspective, despite their significant contribution to overall food loss and waste. Together, these factors highlight the substantial availability and heterogeneous nature of root vegetable processing wastes and underscore the need for integrated valorization strategies.

In addition, the complex biological matrix of root vegetable wastes—composed of starch, structural carbohydrates, proteins, and minor bioactive compounds—can affect both the release of valuable compounds and the efficiency of biological conversion processes. Consequently, appropriate pretreatment and process selection are often required to address matrix-related limitations and to ensure stable and efficient valorization.

Root vegetables interact strongly with their growing environment and may accumulate inorganic contaminants, including heavy metals, through soil and irrigation water uptake [87,88,89]. This characteristic has implications not only for food safety but also for the processing and valorization of root vegetable residues, as contaminant presence can influence downstream treatment options, process selection, and regulatory compliance [87].

These considerations highlight the importance of accounting for feedstock quality and compositional variability when designing sustainable and robust valorization routes for root vegetable processing wastes.

#### 3.1.2. Main Types of Processing Residues

In industrial processing, a substantial portion of root vegetables is transformed into by-products such as peels, trimmings, pomace, pulps, wash waters, and off-grade roots. Depending on the crop type and processing technology, approximately 10–50% of the initial biomass can result in peel and trimming residues [80,90,91]. Pomace and pulps generated from juice, sugar, and starch production can represent 25–50% of the processed root vegetable mass and constitute one of the most voluminous residue streams. In addition, up to 30% of harvested root vegetables may be discarded due to size irregularities, mechanical damage, or cosmetic defects, with only a limited fraction currently redirected to animal feed [92,93]. In industrial vegetable processing, peeling operations represent a major source of solid waste generation. Depending on the crop and processing conditions, peeling can result in substantial biomass losses, with both the quantity and quality of residues being strongly influenced by processing parameters such as temperature, treatment time, and mechanical intensity. These residues, commonly generated as peels and surface trimmings, often retain significant amounts of carbohydrates and bioactive compounds, highlighting their potential for further valorization rather than disposal [94]. An overview of the main processing residues generated from major root vegetables, together with their typical compositional characteristics and relevance for integrated valorization, is summarized in Table 1.

These characteristics underline both the potential and the current underrepresentation of root vegetable processing wastes in integrated valorization studies, providing a strong rationale for examining pigment-rich root vegetable residues in greater detail [112].

At the same time, growing evidence indicates that agri-food by-products often contain equal or higher concentrations of bioactive compounds than edible fractions, particularly in seeds and peels, where phenolic compounds and flavonoids are preferentially accumulated [71]. This dual challenge—environmental burden on the one hand and untapped biochemical potential on the other—has driven increasing interest in sustainable valorization strategies that transform low-value residues into functional materials and energy carriers. However, despite this growing interest, integrated approaches that combine material recovery, complementary energy production, and environmental assessment remain limited, particularly for root vegetable and pigment-rich agri-food wastes.

Such compositional and quality-related aspects need to be considered when selecting appropriate valorization pathways within integrated biorefinery systems.

Root vegetable processing wastes are characterized by high availability and compositional heterogeneity, which directly influence their suitability for different valorization pathways. Understanding the nature of these residues is therefore essential for selecting appropriate cascading strategies within integrated biorefinery systems.

### 3.2. Pigment-Rich Root Vegetable Wastes

**Figure 5:**
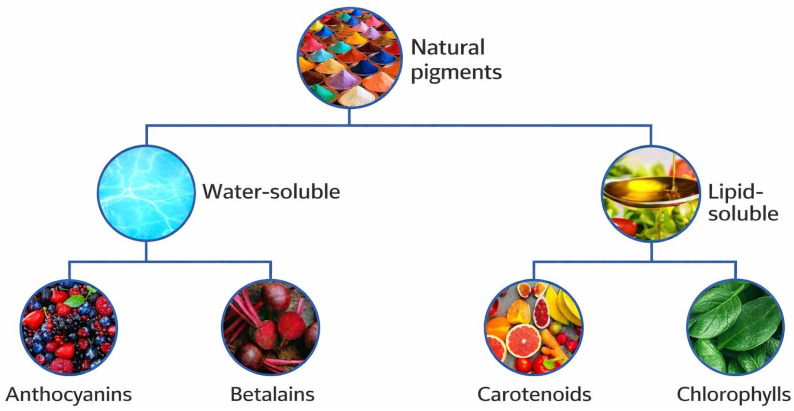
Major classes of plant-derived pigments relevant for waste valorization, classified according to solubility and chemical structure [48].

Current research on plant-derived pigments has primarily focused on compound classes such as anthocyanins, betalains, carotenoids, and chlorophylls (Figure 5), reflecting their abundance and relevance in food and agricultural residues [113,114].

Among these compounds, anthocyanins have attracted particular attention due to their widespread occurrence in fruits and vegetables, high structural diversity, and pronounced sensitivity to environmental conditions. Their molecular structure enables reversible transformations under varying pH conditions, resulting in distinct color changes across the visible spectrum. This behavior contributes to the functional versatility and high-value potential of anthocyanins, particularly when recovered from pigmented agri-food waste streams. Processing purple carrots, purple potatoes, and red cabbage, including sorting and trimming, is the main source of anthocyanin-heavy food discards, while beetroot generates betalains [54,64,65,72,115,116]. These residues represent an attractive feedstock for biorefinery concepts due to their high content of natural pigments with applications in the food, cosmetic, and pharmaceutical industries.

Anthocyanins are water-soluble flavonoids whose stability is highly dependent on pH, temperature, and exposure to light [115]. Consequently, extraction processes are often conducted under mild conditions, frequently using aqueous or hydroalcoholic solvents. After pigment recovery, the remaining biomass retains a substantial fraction of carbohydrates and fibers, enabling further valorization through biological conversion.

The presence of residual phenolic compounds may exert inhibitory effects on microbial activity during fermentation processes, including biohydrogen production. Nonetheless, several studies report that appropriate dilution, pretreatment, or microbial adaptation can mitigate these effects [16,17,54,72,117]. This highlights the importance of considering anthocyanin extraction and energy recovery as sequential, rather than competing, steps within an integrated valorization strategy.

Plant-derived pigments represent a diverse group of compounds that can be broadly classified into water-soluble and lipid-soluble fractions, including anthocyanins, betalains, carotenoids, and chlorophylls [118]. These pigment classes differ substantially in chemical structure, physicochemical behavior, and biosynthetic pathways, which influence their stability, extractability, and suitability for different valorization routes [65]. Among these pigments, anthocyanins and betalains are of particular relevance for root vegetable waste valorization, as they are abundant in processing residues from pigmented vegetables and root crops. Growing interest in these compounds reflects both their functional properties and increasing demand for naturally derived food ingredients. Accordingly, this section focuses on pigment-rich rooted wastes, with emphasis on the pigments most frequently investigated in recent years and their role within integrated valorization strategies.

#### 3.2.1. Sources, Chemical Characteristics, and Stability

Anthocyanin-rich root vegetable wastes are primarily generated from the processing of pigmented fruits and vegetables, including purple carrot, and leaves from red cabbage residues [16]. In these crops, anthocyanins are mainly located in outer tissues such as peels, skins, and pomace, which are commonly discarded during industrial processing. As a result, processing by-products often contain equal or higher concentrations of anthocyanins compared to edible fractions, making them particularly attractive targets for recovery within cascading valorization strategies. The availability of such residues is strongly influenced by regional consumption patterns, processing intensity, and seasonal production, leading to heterogeneous but substantial waste streams across agri-food supply chains.

Their ability to impart red, purple, and blue hues, together with growing consumer demand for naturally derived additives, has positioned anthocyanins as priority targets for recovery from agri-food waste streams [16]. Several root vegetables are notable for their content of naturally occurring pigments with antioxidant properties. While orange carrot cultivars dominate global markets, purple carrot varieties contain high concentrations of anthocyanins. Similarly, beetroot is typically associated with deep-red pigmentation, although yellow and other colored varieties are also cultivated, and is recognized as one of the most pigment-rich vegetables [73]. Anthocyanins and other phenolic compounds present in pigmented root vegetables contribute to their functional value and influence downstream processing options. Processing residues derived from beetroot and purple carrot, therefore, represent important sources of natural colorants and bioactive compounds [3,48]. Consequently, these waste streams are increasingly prioritized for high-value material recovery prior to secondary valorization through biological or biochemical conversion routes.

Anthocyanins belong to the flavonoid family of phenolic compounds and are characterized by a C6–C3–C6 backbone structure. Their chemical behavior is strongly influenced by environmental conditions such as pH, temperature, oxygen exposure, and light, which affect color expression and stability [119].

In agri-food waste matrices, anthocyanins may occur in free or bound forms, interacting with polysaccharides, proteins, and other phenolic compounds. These interactions can influence extraction efficiency and downstream processing performance [54,72]. While anthocyanins are generally considered less stable than other pigment classes, their high functionality and market value justify their prioritization as a first-step recovery option in integrated biorefinery systems. A large range of colors can be derived from anthocyanins (red, orange, blue or purple) according to vegetable sources. Natural pigments from plant biomass are increasingly valued for their functional properties in agri-food systems.

#### 3.2.2. Relevance for Integrated Valorization Systems

Food waste streams derived from fruits and vegetables have attracted increasing attention due to their high content of bioactive compounds, including naturally occurring pigments. These pigments are of particular relevance in agri-food waste valorization, as they combine functional properties with the growing demand for natural food ingredients [48]. While the extraction of bioactive pigments has been extensively investigated, particularly in the context of food and packaging applications, their role within integrated waste valorization systems remains less explored. From a circular biorefinery perspective, pigment recovery is most relevant as a primary, high-value step, after which the remaining biomass can be directed towards complementary valorization pathways, including biological energy recovery [120,121,122,123].

Table 2 reports pigment classes and waste types depending on cultivar, processing technology, and regional practices.

In integrated valorization frameworks, pigment-rich residues offer the advantage of enabling a cascading utilization approach in which high-value compounds are recovered prior to bulk material or energy conversion. After pigment extraction, the remaining biomass typically retains significant amounts of carbohydrates and organic matter, allowing its subsequent use in processes such as anaerobic digestion, fermentation, or composting [3,31,48,57,60]. This sequential use of biomass resources improves overall resource efficiency and reduces the environmental burden associated with single-route waste treatment. Consequently, pigment-rich agri-food wastes represent particularly suitable feedstocks for circular biorefinery systems aimed at combining material recovery with complementary energy production.

These characteristics highlight the potential of pigment-rich agri-food residues as strategic feedstocks for integrated biorefinery concepts, where the recovery of high-value compounds can be combined with subsequent material and energy valorization pathways.

## 4. Valorization Options Within Integrated Biorefinery Systems

The heterogeneous composition of root vegetable wastes requires valorization strategies that go beyond single-output technologies. Integrated biorefinery concepts enable the sequential and complementary use of biomass, prioritizing the recovery of high-value compounds before energy or material recovery from residual streams. Such cascading approaches are particularly relevant for non-lignocellulosic biomass, where mild processing conditions can preserve both economic and environmental value.

**Figure 6:**
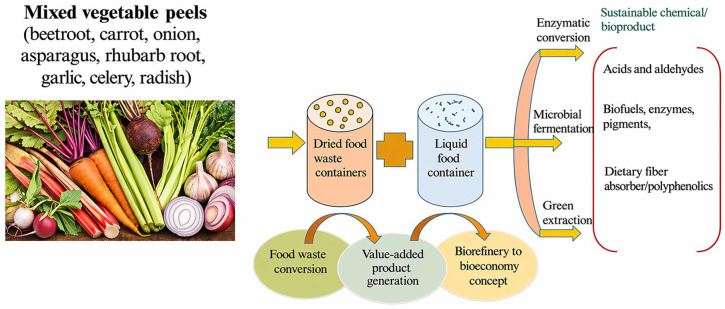
Schematic representation of integrated biorefinery pathways for the valorization of processing wastes (Adapted from [123]).

Integrated biorefinery systems enable the simultaneous conversion of heterogeneous agri-food residues into multiple value-added products through coordinated pretreatment, biochemical conversion, and green extraction pathways. The overall conceptual framework of such integrated valorization routes is illustrated in Figure 6.

The biorefinery paradigm has emerged as a strategic pathway for transforming heterogeneous agro-residues into diversified product streams while improving resource efficiency. Rather than relying on single-product conversion routes, integrated systems combine physicochemical pretreatment, enzymatic hydrolysis, fermentation, and thermochemical upgrading to enable multi-output valorization of lignocellulosic biomass [51,134,135,136]. Recent hybrid thermo–biochemical demonstrations indicate that cascading schemes—such as coupling gasification or pyrolysis with microbial and catalytic upgrading—can enhance carbon recovery and system flexibility [137,138]. Nevertheless, improvements in overall performance remain closely linked to advances in strain engineering, consolidated bioprocessing, and integrated pretreatment strategies [139,140]. Although reported gains in sugar release and microbial productivity are promising, scaling challenges, energy integration, and economic robustness continue to shape the feasibility of advanced lignocellulosic biorefineries [135].

### 4.1. Recovery of High-Value Compounds

These wastes are particularly rich in natural pigments and antioxidant molecules with demonstrated potential for nutraceutical and biocolorant applications [51]. For example, anthocyanins are natural pigments widely used as colorants and antioxidants in food, cosmetic, and pharmaceutical applications [48,54]. Wastes derived from purple carrot and purple potato are particularly rich in these compounds [116]. Extraction processes typically employ aqueous or hydroalcoholic solvents under mild temperature and pH conditions in order to preserve pigment stability. As anthocyanins are sensitive to thermal degradation, their recovery is best positioned at the early stages of biomass processing [54]. In addition to anthocyanins, many root vegetable residues contain significant quantities of polyphenolic compounds with antioxidant and antimicrobial properties. These compounds are often co-extracted with pigments or recovered through targeted extraction processes [64,141]. While polyphenols contribute substantially to the economic value of the extracted fraction, their presence in untreated biomass may inhibit downstream biological conversion processes, further reinforcing the importance of their early removal.

Beyond direct compound extraction, biochemical conversion pathways can further support residue valorization. Anaerobic digestion of mixed wastes has been shown to generate volatile fatty acids and biogas, illustrating complementary recovery of energy carriers alongside biochemical products [51,142]. Recent advances in nanobiotechnology and integrated processing strategies conducted by Lateef et al. [143] have significantly enhanced biomass conversion efficiency, improving the yields of both biofuels and bioactive compounds derived from agricultural wastes. Within this broader valorization framework, anaerobic digestion remains a mature platform capable of converting organic wastes into biogas and biofertilizers while contributing to nutrient recycling in circular bioeconomy systems [144].

Overall, the recovery of high-value compounds not only generates economically attractive products but also modifies the composition of the residual biomass, influencing its subsequent valorization potential. Non-edible fractions of biomass wastes are often particularly rich in polyphenols, polysaccharides, and structural biopolymers, which exhibit antioxidant activity and functional properties relevant for food, material, and environmental applications [145,146]. Within integrated biorefinery concepts, such nonenergy valorization routes can complement bioenergy recovery by prioritizing material extraction and functional use of biomass before residual streams are directed towards biological conversion processes.

Table 3 synthesizes representative extraction and treatment strategies applied to root vegetable and pigment-rich agri-food residues, highlighting the recovered bioactive compounds, potential applications and indicative carbon footprint values.

The examples provided in Table 3 highlight that the environmental performance of recovery processes varies considerably across feedstocks and extraction techniques, confirming the importance of selecting appropriate processing routes within integrated valorization systems. Marić et al. [163] examined the effect of lyophilization compared to heat-based drying method for preserving nutrients in celery, carrot, fennel, purple carrot, parsley, and yellow carrot root vegetables. By analyzing color, antioxidant activity, vitamin C, β-carotene and polyphenols using artificial neural network models, the findings showed that the lyophilization is the superior method and less destructive of the original properties of the analyzed root vegetables than conventional drying. While conventional drying at 50 °C or 70 °C is faster and cheaper, it typically led to significant color changes and a loss of antioxidant characteristics.

### 4.2. Integrated Valorization of Post-Extraction Residues

Following the extraction of pigments, polyphenols, or oils, the remaining biomass typically undergoes significant compositional changes [70,164,165]. Post-extraction residues are generally characterized by: (i) a higher relative carbohydrate content, due to the removal of soluble bioactive fractions; (ii) a reduced concentration of inhibitory compounds, such as phenolics and lipids; and (iii) an improved accessibility of fermentable substrates. These characteristics make post-extraction residues suitable candidates for secondary valorization through biological and biochemical conversion pathways.

Several valorization routes for post-extraction residues have been proposed and investigated in the literature [43,63,166,167]. Biohydrogen production via dark fermentation represents one option for recovering energy from readily fermentable carbohydrate fractions, particularly in residues derived from root vegetables and pigmented crops. Anaerobic digestion is a more established pathway, offering robust conversion of a wider range of organic compounds into biogas and enabling nutrient recycling through digestate application [63]. Alternative routes, such as bioethanol production, have also been explored, particularly for starch-rich residues, although they often require additional hydrolysis steps and may exhibit higher process complexity [168,169]. Composting remains a widely applied reference option for post-extraction residues, providing organic matter stabilization and soil amendment but limited energy recovery [170].

**Figure 7:**
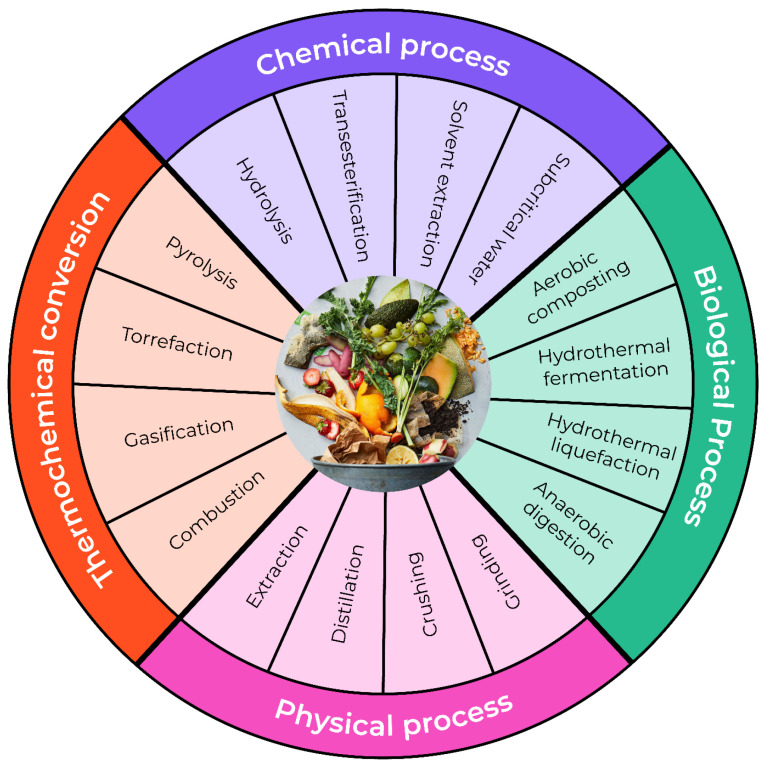
Conceptual framework of integrated circular biorefinery pathways for food waste valorization.

Such an approach facilitates the sequential recovery of high-value compounds, bio-based chemicals, energy carriers, and soil amendments, thereby maximizing carbon efficiency and minimizing residual waste streams [63]. The conceptual structure of these interconnected pathways is illustrated in Figure 7, which highlights the diversity of transformation routes and their role within an integrated circular biorefinery framework.

This integrated perspective provides the conceptual basis for the assessment of biohydrogen as a secondary valorization route and for the life cycle evaluation of alternative system configurations, as discussed in the following sections.

Table 4 provides an overview of valorization options for root vegetable and pigment-rich agri-food wastes in the European Union, with emphasis on cascading use, process limitations, and implications for integrated biorefinery design and life cycle performance.

**Figure 8:**
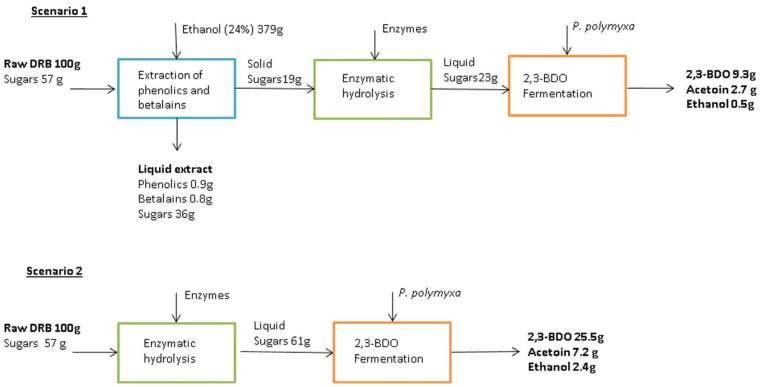
Discarded red beetroot valorization routes for producing bioactive compounds and 2,3-butanediol (Scenario 1), and 2,3-butanediol production only (Scenario 2) [175].

Barrios et al. [175] investigated how discarded red beetroot (DRB) can be converted into multiple value-added products within integrated biorefinery schemes—Figure 8.

A first processing stage enables recovery of ~0.9 g phenolics and ~0.8 g betalains/100 g dry DRB. The remaining solid fraction after extraction can be used for enzymatic hydrolysis and fermentation using *Paenibacillus polymyxa* to produce 2,3-butanediol (2,3-BDO), a valuable platform chemical. In Scenario 2, the entire DRB stream was processed via enzymatic hydrolysis followed by fermentation with *P. polymyxa*, resulting in a global 2,3-BDO yield of 25.5 g per 100 g DRB. Economic assessment revealed that, despite Scenario 2 presenting production costs (10.8 €/kg 2,3-BDO) four times lower than Scenario 1 (42.1 €/kg 2,3-BDO), a multi-product biorefinery configuration (Scenario 1) may represent the most cost-effective option for DRB valorization, as it enables minimum selling prices that are competitive with petrochemical-based production routes [175].

The valorization of beetroot by-products offers a sustainable method to produce third-generation (3G) snacks with enhanced nutritional and functional value [57]. The simple pretreatment consists of the freeze-drying of beetroot waste in a grinder to obtain a free-flowing powder. These snacks are typically made using a corn-based matrix enriched with beetroot by-products and processed via indirect expansion through extrusion operating at the temperatures of the barrel sections of 30/60/100/120 °C and a pressure in the range of 5 bar to 40 bar, and subsequent microwave or heat expansion. To ensure commercial viability, researchers recommend a mixture containing 25% water content and 10% beetroot by-product, achieving the optimal balance of expansion, crunchiness, and functional value. From an operational standpoint, upcycling beetroot by-products, which account for approximately 40% in the process of obtaining liquefied beetroot for atomization, directly supports a circular economy. This process reduces environmental impact by diverting food waste from landfills and adds significant economic value to otherwise discarded materials, allowing manufacturers to market “clean label” products that appeal to eco-conscious consumers.

Mirheli and Dinani [176] demonstrated that combining ultrasonic pretreatment with shaking incubation significantly enhances the extraction of β-carotene from carrot processing waste. Thus, increasing ultrasonic time from 0 to 80 min led to a 73.73% increase in β-carotene content, rising from 30.07 ± 2.08 ppm to 52.24 ± 1.27 ppm.

Consequently, integrated circular biorefinery configurations establish a systemic foundation for optimized carbon recovery and multi-product generation, while simultaneously enabling the development of advanced bioenergy vectors such as biohydrogen.

### 4.3. Case Studies of Biorefineries Processing Root Vegetable Waste

Industrial-scale valorization of food waste into bio-based materials is increasingly gaining attention. For instance, Rowan Minkley and Rob Nicoll, designers based in London, established the initiative Chip[s] Board to produce bioplastics from industrial food waste streams unsuitable for human consumption [177]. In collaboration with McCain Foods, a globally recognized family-owned company specializing in potato-based products, this approach enables the development of durable, recyclable, biodegradable materials without the use of harmful chemicals.

Glycerol and wastewater generated during potato processing represent other low-value by-products with limited recycling potential. A wastewater treatment facility in Pennsylvania facing operational challenges due to these waste streams implemented the Probiotic Solutions Bio Energizer over a two-month period. By enhancing microbial activity, the system achieved reductions in filamentous growth and foaming, improved settling performance, and decreased sludge handling and transportation costs. Initially, the plant experienced loading increases of up to 26% due to these wastes, which led to significant process control difficulties [157].

Recently, Almeida et al. [84] provided a study including a simulated industrial-scale anaerobic digestion system designed to handle 1.60 Mg per day of potato peel and potato offcuts resulting from the potato chip industry, featuring a 165 m^3^ reactor with a 12-day residence time. This scale-up aims to optimize methane production and reduce carbon emissions, with a projected impact of 542 kg of CO_2eq_ per Mg of dry-basis potato peel.

A field-scale experiment was conducted in northern Italy, in the Po Valley, within the framework of the SAFIR Project, to evaluate the impact of treated wastewater reuse on potato yield, quality, and hygiene. The study assessed the presence of *Escherichia coli* and heavy metals in treated wastewater, soil, and tubers. The results indicated that water reuse did not affect sugar levels or dry matter content in the tubers, while the gross margin of the crop increased by up to €765 per hectare per year [77].

The AMWS company from Poland successfully demonstrated that carrot pomace generated as a by-product of industrial carrot mousse production in Tymbark is a suitable and promising feedstock for saccharide production in biochemical and biorefinery applications. The study showed that a dual-stage processing approach offers an effective pathway for improving sugar yield while limiting the formation of degradation products [178]. Specifically, aqueous extraction performed under optimal technological parameters (temperature of 70 °C, extraction time of 60 min, and 5 wt% biomass on a dry basis) yielded 400.2 ± 0.7 mg/g of total saccharides. Subsequent acid-catalyzed hydrolysis of the extraction residue (1 wt%, 150 °C, pH 2, and 90 min) produced an additional 154.4 ± 0.3 mg/g of saccharides. The authors concluded that extending the extraction time beyond 60 min does not provide a significant practical benefit. Also, increasing the pomace loading reduces the solvent-to-solid ratio, limiting the solvent availability per gram of biomass and negatively affecting extraction efficiency. In this context, the removal of readily soluble sugars during the initial extraction step minimizes their degradation during subsequent hydrolysis. This allows the acid treatment to more effectively target structural carbohydrates. As a result, the combined extraction–hydrolysis process achieves higher overall saccharide recovery with reduced degradation compared to single-stage hydrolysis.

Alherbawi et al. [179] demonstrated that pyrolysis, when properly optimized, is a promising approach for the valorization of food waste, including root vegetable residues such as carrot peels and pulp. The study highlighted that the process conditions significantly influence product yields, with higher temperatures favoring bio-oil production while reducing biochar output. However, increased temperature and moisture content also lead to higher energy consumption, making pretreatment steps critical for efficiency. From an economic perspective, the process was found to be most viable at a moisture content of 5% and an operating temperature of 300 °C, resulting in a return on investment of approximately 29% and a payback period of around 3.4 years. Environmentally, pyrolysis offers advantages over conventional waste disposal methods, although impacts such as water use depend on operating conditions. Joensuu et al. [180] conducted a case study on carrot waste in Finland, comparing two valorization approaches: the production of artisan bar soap and the retail of second-class carrots. Their findings indicate that the most effective strategies for food waste management prioritize prevention and the direct use of food for human consumption. The authors developed a six-indicator framework that integrates environmental, economic, and social dimensions, highlighting scalability and waste reduction potential as key determinants of overall impact. The case study demonstrated that simple solutions, such as selling second-class vegetables, outperform more innovative valorization approaches like cosmetic production, which are often limited in scalability and contribute less significantly to reducing food waste.

Another study [181] demonstrated that the environmental performance of hydrochar production from the valorization of sugar beet pulp, a root crop residue, is largely determined by the drying method applied after hydrothermal carbonization. The results show that energy-intensive techniques such as freeze-drying and oven-drying generate significantly higher environmental impacts, while natural and solar drying methods offer more sustainable alternatives.

A life cycle assessment (LCA) was conducted to evaluate two processing routes for converting carrot residues into cellulose nanofibres at pilot scale, with the aim of providing eco-design insights and assessing environmental performance for potential industrial applications [182]. The first route involved washing, alkali treatment, and bleaching, while the second route relied solely on bleaching. The functional unit and system boundaries were defined as 1 kg of a 2 wt% carrot nanofiber suspension, corresponding to a dry mass of 20 g. The results indicated that the life cycle cost for producing 1 kg of suspension via the first route was 0.90 €, whereas comparatively, the second route achieved a significantly lower cost of 0.42 €, representing a reduction of about 50%. In addition to economic benefits, the simplified bleaching-only route led to a 75% reduction in environmental impacts. By removing the alkali pretreatment step, a substantial improvement in process efficiency was achieved, more than doubling the yield to 65% while maintaining high-quality nanofibers suitable for bio-based applications.

**Figure 9:**
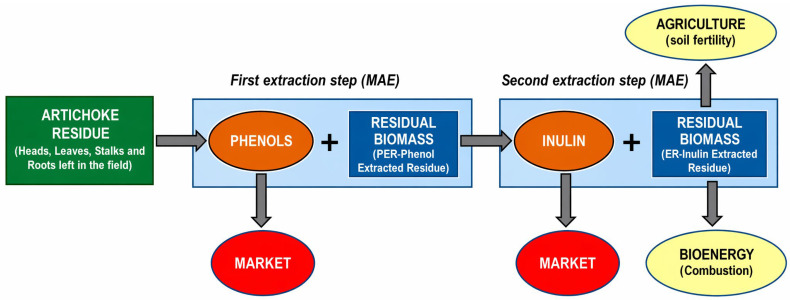
A sequential biorefinery-based process designed to valorize artichoke plant residues left in the field [105].

A sequential processing strategy based on a biorefinery concept was developed in Italy for the valorization of artichoke field residue—Figure 9.

Two cascading, eco-friendly extraction processes based on microwave-assisted extraction (MAE) and the use of green solvents (water and ethanol) were optimized for the valorization of artichoke biomass [105]. In the first stage, phenolic compounds are extracted from the raw material, yielding a marketable phenolic extract and a by-product referred to as phenol extracted residue (PER). In the second extraction stage, PER is further processed to recover polysaccharides, producing an additional high-value product, namely inulin, along with a secondary by-product (inulin-extracted residue, IER). The resulting IER was subsequently characterized to assess its potential for further valorization, particularly as a bioenergy feedstock and as a soil amendment in agricultural applications (e.g., green manure). However, the scalability of the integrated process remains to be addressed in future research.

Despite these notable industrial- and field-scale applications, the literature still contains relatively few fully implemented biorefinery case studies at an industrial scale. Most existing research remains concentrated at the laboratory or pilot scale, highlighting the need for further experimental advancements to support the transition toward large-scale deployment of biorefinery concepts. The majority of the processes discussed in this section are currently at TRL 3–6, corresponding to experimental validation at laboratory scale and, in some cases, pilot-scale demonstration. Only a limited number of technologies have progressed to higher TRL levels (TRL 7–9), which involve system demonstration in operational environments or full commercial deployment. This relatively low level of technological maturity explains the scarcity of documented full-scale industrial biorefineries in the EU within the scientific literature. In many cases, existing initiatives are still under development, funded through European projects, or reported primarily in technical reports and industrial communications rather than peer-reviewed publications. Therefore, the analysis presented here reflects the current state of development of the field, which is still transitioning from research to industry.

## 5. Research Gaps and Future Perspectives

Despite the growing body of literature on agri-food waste valorization, several critical research gaps remain, particularly concerning the waste categories addressed in this review.

First, experimental data on root vegetable and anthocyanin-rich waste streams remain fragmented, with many studies focusing on single feedstocks under laboratory conditions. There is a clear need for pilot-scale investigations that account for feedstock variability, seasonal availability, and realistic process integration.

Second, the interaction between bioactive compounds and biological conversion processes is not yet fully understood. Residual phenolics and pigments may inhibit microbial activity during fermentation, yet their impact is often insufficiently quantified or overlooked in system-level assessments.

Third, from an environmental-assessment perspective, LCA studies specifically addressing integrated biorefineries for these waste streams are scarce.

Current research on the valorization of root vegetable and anthocyanin-rich agri-food wastes remains largely fragmented, with most studies focusing on isolated technological or environmental aspects. Future research would benefit from the adoption of a holistic framework that simultaneously addresses technical, economic, and social dimensions of integrated waste management systems.

From a technical perspective, there is a clear need for improved characterization of agri-food waste streams, including their spatial and temporal availability, compositional variability, and realistic production capacities at regional and national scales. While laboratory-scale studies provide valuable insights, limited attention has been given to inventory-based assessments, system integration, and decision-support tools that could guide the implementation of cascading biorefinery concepts under real operating conditions.

From an economic perspective, the value of agri-food waste valorization pathways remains insufficiently quantified beyond individual process outputs. Although high-value compounds and bioenergy carriers can generate added value along the supply chain, comprehensive assessments of production costs, market dynamics, and downstream value creation are still scarce. This limits the ability to compare alternative valorization routes and to support investment and policy decisions.

From a social and societal perspective, the broader implications of agri-food waste valorization are rarely addressed.

Addressing these gaps will be essential for advancing the practical implementation of sustainable, integrated valorization strategies for agri-food wastes.

Beyond material recovery and energy-oriented pathways, anthocyanins recovered from agri-food wastes have also been explored in niche downstream applications, such as intelligent packaging and visual freshness indicators. However, these applications remain highly technology-specific and fall outside the scope of integrated biorefinery systems, where the primary focus lies on cascading valorization and system-level environmental performance.

Recent research increasingly emphasizes the cascading valorization of agri-food wastes, integrating the recovery of high-value compounds with subsequent biological conversion processes—such as dark fermentation-based biohydrogen production and anaerobic digestion—to enhance resource efficiency and reduce environmental impacts.

## 6. Conclusions

This review has examined the valorization potential of wastes derived from root vegetables and anthocyanin-rich wastes processing within a circular biorefinery perspective. These waste streams exhibit diverse but complementary characteristics, enabling their integration into multi-output systems that combine material recovery, energy production, and nutrient recycling.

The extraction of high-value compounds represents the primary and most economically attractive valorization step for several of the waste streams addressed in this review. Pigmented vegetable wastes, including those derived from root wastes, contain bioactive and functional compounds with established market demand, making their recovery a key driver for biorefinery feasibility.

The heterogeneous nature of wastes derived from root vegetables, anthocyanin-rich crops, and agri-food waste processing necessitates a systems-oriented valorization approach rather than isolated technological solutions. Integrated circular biorefinery concepts enable the sequential recovery of high-value compounds, followed by complementary energy generation and nutrient recycling, thereby maximizing overall resource efficiency.

In integrated biorefinery systems, the selection of post-extraction valorization routes is highly context-dependent and influenced by local infrastructure, energy demand, and environmental performance. For this reason, comparative assessment tools, such as life cycle assessment, are essential to identify optimal valorization pathways.

The evidence reviewed suggests that integrated valorization pathways consistently outperform single-route approaches, both in terms of resource efficiency and environmental performance. By adopting a holistic, life cycle-based perspective, agri-food waste management strategies can move beyond disposal-oriented practices towards genuinely circular solutions.

## Figures and Tables

**Table 1:** Major processing residues from major root vegetables and their compositional characteristics relevant for integrated valorization.

Root Vegetable	Main Processing Residues	Typical Share of Biomass	Key Compositional Features	Valorization Relevance	References
Potato	Peels, off-grade tubers, starch-rich wash waters	Peels: 15–40% Rejects: up to 30%	High starch content, soluble sugars, dietary fiber, proteins; low lignin	Starch recovery; dark fermentation; anaerobic digestion; cascading biorefinery routes	[77,90,91,95,96,97]
Carrot	Peels, pomace, leaves, rejects	Peels & pomace: 20–45% Leaves: 20–30%	Soluble sugars, dietary fibers, carotenoids; purple varieties rich in anthocyanins and polyphenols	Pigment extraction; fermentation-based energy recovery; composting	[46,47,68,93,98]
Beetroot	Peels, pomace, pulp	35–40%	Betalains, phenolic compounds, soluble sugars, dietary fiber	High-value pigment extraction; dark fermentation; integrated biorefinery pathways	[55,56,57,58,99,100,101,102]
Asparagus	Roots and peels	76.5%	Molecular mass of 2.36 × 10^4^ Da; 92.5 % polysaccharides, protein content of 4.23 %, and impurities of 3.27 %. Monosaccharide composition revealed the presence of rhamnose, galacturonic acid, glucose, galactose, xylose, and arabinose in a molar ratio of 6.72:1.76:1.00:38.65:8.10:2.2	A bio-flocculant polysaccharide for removing methylene blue (MB), Rhodamine B (RhB), heavy metals, and suspended particles	[103]
Radish and turnip	Roots and leaves		Glucosinolates, flavonoid glycosides, phenolic acids, organic acids, and fatty acids	In pharma, as antivirulence phytochemicals	[104]
Artichoke	Heads, leaves, stalks, and roots	80–85%	Phenols (36.61 ± 1.28 kg Ha^−1^), Inulin (4602.72 ± 83.09 28 kg Ha^−1^), and solid residue (4.17 ± 0.15 t Ha^−1^)	High-value compounds (phenols, inulin, etc.), bioenergy and agricultural applications	[105]
Endive	Forced roots		Sugars, dietary fibers, phenolic compounds and sesquiterpenes lactones	Dietary fibers	[106]
Celery *	Roots, stem and leaves		Free-radical scavengers from the flavonol group (4.19 mg/100 g d.w., including luteolin (0.81 mg/100 g d.w.) and apigenin (3.39 mg/100 g d.w.) and phenolic acids (caffeic acid, ferulic acid, and *p*-coumaric acid)	In pharma for metabolic parameters, oxidative stress, blood pressure, but potent allergen	[74]
Parsley *	Roots, leaves		High content of K (399 mg/100 g) and Ca (43 mg/100 g), folates (180 µg/100 g)	Antioxidant potential, diuretic effect, decreased depressive behavior	[74]
Black garlic	Roots		S-allyl-L-cysteine (SAC), organosulfur compounds and phenolic compounds	Antioxidant, prebiotic, anti-inflammation, used for N/S/O self-doped carbon dots and biochar	[52,107,108]
Turmeric	Rhizomes	40%	α-phellandrene, p-cymene, eucalyptol, ar-curcumene, zingiberene, ar-turmerone, curlone, and turmerone; curcumin	Essential oil; A reducing agent for eco-friendly synthesis of CuO and ZnO NPs; Food packaging material	[109,110,111]

* Most articles for celery and parsley are focused on leaves.

**Table 2:** Main pigment-rich agri-food waste streams relevant for integrated valorization pathways.

Waste Source	Dominant Pigment Class	Typical Waste Stream	Valorization Relevance	References
Beetroot pomace/peels	Betalains	Juice & slicing residues	Natural colorants; antioxidant extracts	[124,125]
Purple carrot pomace	Anthocyanins	Juice & puree residues	High-value pigment recovery	[66,126]
Red cabbage outer leaves	Anthocyanins	Trimming & discarded leaves	Pigment extraction prior to bioconversion	[117,127]
Mixed vegetable peels	Polyphenols & pigments	Industrial peeling residues	Functional extracts; cascade use	[59,60]
Carrot peels	Carotenoids	Peeling residues	Pigment & antioxidant recovery	[49,59]
Leafy residues (beet, carrot)	Chlorophylls & phenolics	Leaf removal	Limited valorization; pretreatment needed	[128,129]
Onion skins, stalk, roots, petiole, flower, and damaged bulbs	Quercetine, Anthocyanins	Dishes and condiments preparation	Edible coating; active packaging; smart packaging	[130]
Rhubarb root	Anthraquinones	Food, pharmaceuticals and industry	Dietary supplements and natural edible pigments	[131]
Asparagus	Saponin and phenolic acids	Roots and rhizomes discarded during asparagus cultivation	Food, nutraceuticals and agriculture (potential anti-pesticide and biostimulant activity on different crops)	[50]
Chicory (radicchio), red chicory	Lutein, chlorophyll, polyphenols, flavonoids, chlorogenic and caffeic acid, quercetin, and kampferol derivatives	Leaves, roots, and steams	Nutraceutical, food and feed industry	[43]
Parsnip	Phenols, flavonoids, fatty acids, minerals	Rotten roots	Functional nutrients for the elderly	[132]
Leek	Chlorophyll, β-carotene, lutein, flavonoids, pentanol, methyl furan, polysaccharides	Residues	Lignocellulose micro- and nanofibril suspensions	[133]

**Table 3:** Recovery routes and indicative carbon footprint of bioactive compound extraction from selected root vegetables wastes.

Wastes	Bioactive Compounds and Applications	Extraction Methods	Carbon Footprint, kg CO_2_e/kg	References
Carrots	Carotenoids for antioxidant activity and natural pigments	Microwave-assisted extraction (MAE); a mixture of extracting solvents: 50% (*v*/*v*) hexane, 25% (*v*/*v*) acetone and 25% (*v*/*v*) ethanol; 180 W microwave power	0.41	[67,147]
Onions	Quercetin as supplements and antioxidant properties, enzymatic inhibitors	Subcritical solvent extraction (SSE) at temperature in the range of 120–140 °C and subcritical water extraction (SWE) at a temperature of 45 °C, semi-continuous hydrolysis at temperature in the range of 105–180 °C, pressure of 5 MPa, and time of 180 min	0.44	[148,149]
Garlic	Allicin in food and nutraceutical industries as antimicrobial, anti-inflammatory and antioxidant agent, carbon precursor for lithium-ion batteries	Simultaneous microwave-assisted extraction (SME) at a temperature of 50 °C and time of 3 min; ultrasonic-assisted extraction (UAE) at a temperature of 25 °C and time of 90 min; direct pyrolysis at a temperature of 180 °C	0.39	[52,150,151]
Celery	Phenolic and terpenoid compounds as inhibitors for lipid and protein oxidation from processed foods, enhancing shelf life and product stability	Ultrasound–microwave-assisted extraction (UMAE); deep eutectic solvents: 1,2-propanediol and choline chloride; 30% (*w*/*w*) water content, a solid–liquid ratio of 1/35.33 g/mL, 5 min ultrasound time, 20 °C ultrasound temperature, 600 W ultrasound power, microwave time and intensity: 4.73 min and 123 W/mL, respectively	0.08	[152,153]
Beetroot and beet sugar	Betalains, phenolics, vitamins, and antioxidants for replacing synthetic additives, food and pharma industries; pectin for food, pharmaceutical, and cosmetic industries	MAE, hydrothermal extraction (HE), conventional solvent extraction (CSE): ratio between beetroot peels:ethanol 50% is 1:10; citric acid at a concentration in the range of 1.5% was used for acidification of the extract; a temperature of 52.52 °C and extraction time of 49.9 min	0.23–0.67	[55,58,61,69,154]
Parsley	Biodegradable films	Alkali treatment at a temperature of 40 °C and time of 1 h	0.61	[155]
Potatoes	Catechins, phenolic acids, and flavonoid compounds for food industry	Ethanol, blanching and ultrasound pretreatment conducted with a power of 400 W, temperature of 25 °C, and time of 20 min	0.29	[53,80,156,157]
Radish	Carbohydrates, enzymes, flavonoids, glucosinolates, organic acids, phenolic compounds, sulfur compounds, polysaccharides as anticancer, antihypertensive, anti-inflammatory, antimicrobial, anti-obesity, antiulcerative, and intestinal motility stimulation activities	Aqueous or solvent extractions	0.17	[158]
Horseradish	Phenolic, antioxidants, flavonoid compounds as functional ingredients in food products	Conventional extraction performed with a magnetic stirrer at 700 rpm, room temperature, and time of 60 min	2.24	[62,159]
Turnip	Flavonoids, phenolic acids, fatty acids, glucosinolates as potential antibacterial and antivirulence compounds for agri-food and pharmaceutical industries	Cold maceration at a temperature of 25 °C, using methanol as solvent followed by vacuum vaporation	0.16	[104,160]
Artichoke	Phenols and inulin with therapeutic properties (blood circulation, mobilization of energy reserves, induction of choleresis, inhibition of cholesterol biosynthesis) and biotechnological applications	Microwave-assisted extractions (MAE) and green solvents (water and ethanol)	0.60	[105,161]
Ginger and turmeric	Phenolic compounds, such as 6-gingerol and its derivatives with antioxidant and antimicrobial activity; 65%/57% protein extracts from ginger and the turmeric wastes	High-intensity focused ultrasounds (HIFUs): 60 mg of dried root waste with 5 mL water; 18 min/25 min, at 60 °C and 80% amplitude; enzyme-assisted extraction (EAE): 300 µL enzyme/mg substrate; subcritical water extraction (SWE): 1:6 ratio between waste to sand; preheating time of 5 min, pressure of 1700 psi; 175 °C for 15 min	0.79 3.77	[162]

**Table 4:** Valorization pathways for root vegetable and pigment-rich agri-food wastes in the EU.

Waste Fraction	Valorization Route	Main Advantages	Key Limitations	Role in Integrated Biorefinery	References
Root vegetable peels & trimmings (potato, beetroot, carrot)	Extraction of pigments & polyphenols	High economic value; alignment with EU bioeconomy strategy; low processing volumes	Solvent and energy demand; residue management required	Priority step enabling cascading use	[61,167]
Pigment-rich wastes (beetroot, purple carrot, red cabbage residues)	Recovery of anthocyanins/betalains	Strong market demand for natural colorants; food & cosmetic applications	Stability issues; sensitivity to pH and temperature	High-value material recovery before energy routes	[61,167]
Post-extraction carbohydrate-rich residues	Dark fermentation (biohydrogen)	Rapid energy recovery; suitable for wet wastes; complementary to anaerobic digestion (AD)	Low H_2_ yields; process instability; microbial competition	Intermediate energy recovery step	[42,167,171,172]
Fermentation effluents & mixed residues	AD (biogas)	Mature technology in EU & RO; grid injection and combined heat and power (CHP) compatibility	Lower energy density than H_2_; longer retention times	Stabilization and maximization of energy recovery	[51,63,123,167,173]
Digestate	Agricultural application/soil amendment	Nutrient recycling; compliance with EU circular economy goals	Quality control; contaminant regulations	Closing material loops	[123,172,174]
Mixed agri-food wastes (baseline)	Composting	Low cost; regulatory acceptance	Limited energy recovery; lower economic value	Reference scenario for LCA	[51,123,172]

## Data Availability

No new data were created or analyzed in this study. Data sharing is not applicable to this article.

## References

[1] Râpă M., Darie-Niță R.N., Coman G. (2024). Valorization of fruit and vegetable waste into sustainable and value-added materials. Waste.

[2] Zhu Y., Luan Y., Zhao Y., Liu J., Duan Z., Ruan R. (2023). Current technologies and uses for fruit and vegetable wastes in a sustainable system: A review. Foods.

[3] Hrelia S., Angeloni C., Barbalace M.C. (2023). Agri-Food wastes as natural source of bioactive antioxidants. Antioxidants.

[4] Matei E., Râpă M., Predescu A.M., Țurcanu A.A., Vidu R., Predescu C., Bobirica C., Bobirica L., Orbeci C. (2021). Valorization of agri-food wastes as sustainable eco-materials for wastewater treatment: Current state and new perspectives. Materials.

[5] Moraes N.V., Lermen F.H., Echeveste M.E.S. (2021). A systematic literature review on food waste/loss prevention and minimization methods. J. Environ. Manag..

[6] Roy P., Graceffa V. (2024). From waste to therapeutics: Extraction methodologies and biological properties of bioactive compounds from fruit and vegetable waste. Food Biosci..

[7] Ishangulyyev R., Kim S., Lee S.H. (2019). Understanding food loss and waste—Why are we losing and wasting food?. Foods.

[8] Calabró G., Vieri S. (2025). Food waste and the EU target: Effects on the agrifood systems’ sustainability. J. Foodserv. Bus. Res..

[9] Centre J.R. (2023). Less Food Waste Could Bring Lower EU Food Prices and Decrease Greenhouse Gas Emissions. https://joint-research-centre.ec.europa.eu/jrc-news-and-updates/less-food-waste-could-bring-lower-eu-food-prices-and-decrease-greenhouse-gas-emissions-2023-07-06_en.

[10] Eurostat 130 kg of Food Wasted per Person Annually in the EU. https://ec.europa.eu/eurostat/web/products-eurostat-news/w/ddn-20251016-2.

[11] Eurostat How Much Fruit and Vegetables Does the EU Harvest? 2025. https://ec.europa.eu/eurostat/en/web/products-eurostat-news/w/ddn-20250825-1.

[12] Be the Story Food Waste: What Are the Most Wasted Foods?. https://www.be-the-story.com/en/food-waste/food-waste-what-are-the-most-wasted-foods/.

[13] Eurostat Agricultural Production. https://ec.europa.eu/eurostat/statistics-explained/index.php?title=Agricultural_production_-_crops.

[14] UkrAgroConsult (2025). Which EU Countries Harvest the Most Vegetables and Fruits. https://ukragroconsult.com/en/news/which-eu-countries-harvest-the-most-vegetables-and-fruits/.

[15] Berti G. (2020). Sustainable agri-food economies: Re-territorialising farming practices, markets, supply chains, and policies. Agriculture.

[16] Oliveira J., Benvenutti L., Albuquerque B.R., Finimundy T.C., Mandim F., Pires T.C., Pereira C., Corrêa R.C.G., Barros L., Zielinski A.A.F. (2025). Green extraction of anthocyanin from red cabbage waste using acid whey as a promising bio-based solvent. Innov. Food Sci. Emerg. Technol..

[17] Benvenutti L., Zielinski A.A.F., Ferreira S.R.S. (2022). Pressurized aqueous solutions of deep eutectic solvent (DES): A green emergent extraction of anthocyanins from a Brazilian berry processing by-product. Food Chem. X.

[18] de Albuquerque B.R., Corrêa R.C.G., de Lima Sampaio S., Barros L. (2023). Bioactive compounds from food and its by-products: Current applications and future perspectives. Food Waste Conversion.

[19] Radulescu D., Neacsu I., Vasile B., Surdu V., Andronescu E. (2025). Green synthesis of copper, zinc, and magnesium oxide nanoparticles using orange peel extract. Univ. Politeh. Buchar. Sci. Bull. Ser. B-Chem. Mater. Sci..

[20] Marcu I., Radu G. (2022). The effect of peel and seed removal on the physical and chemical properties of the tomato fruits (*Solanum lycopersicum* L.). Univ. Politeh. Buchar. Sci. Bull. Ser. B-Chem. Mater. Sci..

[21] https://www.trade.gov/country-commercial-guides/romania-agricultural-products.

[22] Țurcanu A.A., Matei E., Râpă M., Predescu A.M., Berbecaru A.-C., Coman G., Predescu C. (2022). Walnut shell biowaste valorization via HTC process for the removal of some emerging pharmaceutical pollutants from aqueous solutions. Int. J. Mol. Sci..

[23] Moroșan E., Dărăban A., Popovici V., Rusu A., Ilie E.I., Licu M., Karampelas O., Lupuliasa D., Ozon E.A., Maravela V.M. (2024). Socio-demographic factors, behaviors, motivations, and attitudes in food waste management of Romanian households. Nutrients.

[24] Voicila D.N., Sterie C.M., Dragomir V. (2024). Agricultural waste in EU and Romania within the framework of advancing bioeconomic growth. Lucr. Științifice Manag. Agric..

[25] Moroșan E., Popovici V., Popescu I.A., Daraban A., Karampelas O., Matac L.M., Licu M., Rusu A., Chirigiu L.-M.-E., Opriţescu S. (2025). Perception, trust, and motivation in consumer behavior for organic food acquisition: An exploratory study. Foods.

[26] Sterie M.C., Stoica G.D., Giucă A.D., Bogos I.B. (2023). An overview of the vegetable sector in Romania. Proceedings of the Competitiveness of Agro-Food and Environmental Economy.

[27] Ivanovski M., Urbancl D., Petrovič A., Stergar J., Goričanec D., Simonič M. (2022). Improving lignocellulosic and non-lignocellulosic biomass characteristics through torrefaction process. Appl. Sci..

[28] Kannan S., Raghavan V. (2023). Hydrothermal carbonization of nonlignocellulosic wastes using enzyme pretreatment. Value-Addition in Agri-Food Industry Waste Through Enzyme Technology.

[29] Tomeleri J.O.P., Varanda L.D., Pitombo L.M., Yamaji F.M., Pádua F.A.D. (2021). Influence of non-lignocellulosic elements on the combustion of treated wood and wooden panel. Sustainability.

[30] Tye Y.Y., Lee K.T., Abdullah W.N.W., Leh C.P. (2016). The world availability of non-wood lignocellulosic biomass for the production of cellulosic ethanol and potential pretreatments for the enhancement of enzymatic saccharification. Renew. Sustain. Energy Rev..

[31] Roselli V., Pugliese G., Leuci R., Brunetti L., Gambacorta L., Tufarelli V., Piemontese L. (2024). Green methods to recover bioactive compounds from food industry waste: A sustainable practice from the perspective of the circular economy. Molecules.

[32] Saady N.M.C., Hernández A.V., Flores Servin K.L., Rodriguez J.Z., Haque M.A., Owusu M.K., Zendehboudi S., Bazan C., Ruiz Espinoza J.E. (2026). Valorization of Agro-Food Plant Wastes: Bioactive Compound Profiles and Biotechnological Potential of Twenty Crops. Recycling.

[33] Bekavac N., Krog K., Stanić A., Šamec D., Šalić A., Benković M., Jurina T., Gajdoš Kljusurić J., Valinger D., Jurinjak Tušek A. (2025). Valorization of food waste: Extracting bioactive compounds for sustainable health and environmental solutions. Antioxidants.

[34] Greses S., Tomás-Pejó E., Gonzalez-Fernandez C. (2022). Food waste valorization into bioenergy and bioproducts through a cascade combination of bioprocesses using anaerobic open mixed cultures. J. Clean. Prod..

[35] Wolniak R., Grebski W.W. (2025). Analysis of Waste Trends in the European Union (2021–2023): Sectorial Contributions, Regional Differences, and Socio-Economic Factors. Foods.

[36] Ram M., Bracci E. (2024). Waste management, waste indicators and the relationship with sustainable development goals (SDGs): A systematic literature review. Sustainability.

[37] Rodríguez-Jiménez L., Pérez-Vidal A., Torres-Lozada P. (2022). Research trends and strategies for the improvement of anaerobic digestion of food waste in psychrophilic temperatures conditions. Heliyon.

[38] Alli Y., Ejeromedoghene O., Dembaremba T., Adawi A., Alimi O., Njei T., Bamisaye A., Kofi A., Anene U., Adewale A. (2025). Perspectives on the status and future of sustainable CO2 conversion processes and their implementation. Carbon Capture Sci. Technol..

[39] de Paula M., Groeneveld J., Huth A. (2015). Tropical forest degradation and recovery in fragmented landscapes—Simulating changes in tree community, forest hydrology and carbon balance. Glob. Ecol. Conserv..

[40] Murphy D. (2024). Carbon Sequestration by Tropical Trees and Crops: A Case Study of Oil Palm. Agriculture.

[41] Das S., Basak N. (2024). Optimization of process parameters for enhanced biohydrogen production using potato waste as substrate by combined dark and photo fermentation. Biomass Convers. Biorefinery.

[42] Melikoglu M., Tekin A. (2024). Biohydrogen production from food and agricultural wastes: A global review and a techno-economic evaluation for Turkey. Int. J. Hydrogen Energy.

[43] Peron G., Ferrarese I., Carmo Dos Santos N., Rizzo F., Gargari G., Bertoli N., Gobbi E., Perosa A., Selva M., Dall’Acqua S. (2024). Sustainable extraction of bioactive compounds and nutrients from agri-food wastes: Potential reutilization of berry, honey, and chicory byproducts. Appl. Sci..

[44] Arora S., Siddiqui S., Gehlot R. (2019). Physicochemical and Bioactive Compounds in Carrot and Beetroot Juice. Asian J. Dairy Food Res..

[45] El-Saadony M.T., Saad A.M., Mohammed D.M., Alkafaas S.S., Abd El-Mageed T.A., Fahmy M.A., Ezzat Ahmed A., Algopishi U.B., Abu-Elsaoud A.M., Mosa W.F. (2025). Plant bioactive compounds: Extraction, biological activities, immunological, nutritional aspects, food application, and human health benefits—A comprehensive review. Front. Nutr..

[46] Klettenhammer S., Ferrentino G., Zendehbad S.H., Morozova K., Scampicchio M. (2021). Bioactive compounds from carrot pomace as natural antioxidants to enhance the oxidative stability of linseed oil encapsulated by particles from gas saturated solutions technique. Chem. Eng. Trans..

[47] Song R., Ismail M., Baroutian S., Farid M. (2018). Effect of subcritical water on the extraction of bioactive compounds from carrot leaves. Food Bioprocess Technol..

[48] Martins R., Sales H., Pontes R., Nunes J., Gouveia I. (2023). Food wastes and microalgae as sources of bioactive compounds and pigments in a modern biorefinery: A review. Antioxidants.

[49] Ramírez-Pulido B., Bas-Bellver C., Betoret N., Barrera C., Seguí L. (2021). Valorization of vegetable fresh-processing residues as functional powdered ingredients. A review on the potential impact of pretreatments and drying methods on bioactive compounds and their bioaccessibility. Front. Sustain. Food Syst..

[50] Alcaide I., Hamdi A., Guillein-Bejarano R., Jiménez-Araujo A., Rodríguez-Arcos R. (2023). Sustainable valorization of co-products from asparagus cultivation by obtaining bioactive compounds. Front. Plant Sci..

[51] Gautam S., Bora B., Dutta D., Tripathi A., Srivastava J., Thatoi H., Srivastava S., Khade S., Geed S. (2025). Integrated biorefinery approaches for the sustainable valorization of agricultural residues into biofuels, bioplastics, and bioactive compounds. Sustain. Chem. Clim. Action.

[52] Jiménez-Amezcua I., González-Prada A., Díez-Municio M., Soria A.C., Ruiz-Matute A.I., Sanz M.L. (2023). Simultaneous microwave-assisted extraction of bioactive compounds from aged garlic. J. Chromatogr. A.

[53] Santos N.C., Almeida R.L., da Silva L.A., de Lima T.L., Leite M.D.O., Cruz J.P., Dias R.A.D.L., Silva V.M.D.A., de Sousa S., de Araújo S.N. (2026). Valorization of potato waste via pretreatments and drying: Enhancement of bioactive compounds, antioxidant activity, and stability for sustainable applications. Food Bioprod. Process..

[54] Arruda H.S., Silva E.K., Peixoto Araujo N.M., Pereira G.A., Pastore G.M., Marostica Junior M.R. (2021). Anthocyanins recovered from agri-food by-products using innovative processes: Trends, challenges, and perspectives for their application in food systems. Molecules.

[55] Stoica F., Râpeanu G., Rațu R.N., Stănciuc N., Croitoru C., Țopa D., Jităreanu G. (2025). Red beetroot and its by-products: A comprehensive review of phytochemicals, extraction methods, health benefits, and applications. Agriculture.

[56] Sani I.K., Masoudpour-Behabadi M., Sani M.A., Motalebinejad H., Juma A.S., Asdagh A., Eghbaljoo H., Khodaei S.M., Rhim J.-W., Mohammadi F. (2023). Value-added utilization of fruit and vegetable processing by-products for the manufacture of biodegradable food packaging films. Food Chem..

[57] Igual M., Moreau F., García-Segovia P., Martínez-Monzó J. (2023). Valorization of beetroot by-products for producing value-added third generation snacks. Foods.

[58] Lazăr S., Constantin O.E., Stănciuc N., Aprodu I., Croitoru C., Râpeanu G. (2021). Optimization of betalain pigments extraction using beetroot by-products as a valuable source. Inventions.

[59] Newson W.R., Johansson E., Papoutsis K. (2025). Holistic approach in the valorization of fruit and vegetable by-products generated through processing and postharvest storage. Crit. Rev. Biotechnol..

[60] Ficano G., Galdi G., De Sio F., Rapacciuolo M., Sandei L., Cacace D. (2024). “Green” extraction of bioactive molecules from vegetables and fish industry by-products. Environ. Eng. Manag. J. (EEMJ).

[61] Sharma M., Usmani Z., Gupta V.K., Bhat R. (2021). Valorization of fruits and vegetable wastes and by-products to produce natural pigments. Crit. Rev. Biotechnol..

[62] Marković J.M., Salević A.S., Milinčić D.D., Gašić U.M., Đorđević V.B., Rabrenović B.B., Pešić M.B., Lević S.M., Mihajlović D.M., Nedović V.A. (2025). Horseradish (*Armoracia rusticana* L.) Processing By-Products as Potential Functional Ingredients in Food Production: A Detailed Insight into Phytochemical Composition and Antioxidant Properties. Separations.

[63] Pant M., Bisen D., Kewlani P., Srivastav A.L., Bhatt I.D., Chakma S. (2025). Review of food waste valorization technologies: A sustainable approach to resource recovery and utilization. Biomass Futures.

[64] Diaconeasa Z., Iuhas C.I., Ayvaz H., Mortas M., Farcaş A., Mihai M., Danciu C., Stanilă A. (2022). Anthocyanins from agro-industrial food waste: Geographical approach and methods of recovery—A review. Plants.

[65] Rodriguez-Amaya D.B. (2019). Update on natural food pigments-A mini-review on carotenoids, anthocyanins, and betalains. Food Res. Int..

[66] Pérez M.B., Carvajal S., Beretta V., Bannoud F., Fangio M.F., Berli F., Fontana A., Salomón M.V., Gonzalez R., Valerga L. (2023). Characterization of purple carrot germplasm for antioxidant capacity and root concentration of anthocyanins, phenolics, and carotenoids. Plants.

[67] Elik A., Yanık D.K., Göğüş F. (2020). Microwave-assisted extraction of carotenoids from carrot juice processing waste using flaxseed oil as a solvent. LWT.

[68] Kamiloglu S., Ozkan G., Isik H., Horoz O., Van Camp J., Capanoglu E. (2017). Black carrot pomace as a source of polyphenols for enhancing the nutritional value of cake: An in vitro digestion study with a standardized static model. LWT.

[69] Rathee P., Sehrawat R., Rathee P., Khatkar A., Akkol E.K., Khatkar S., Redhu N., Türkcanoğlu G., Sobarzo-Sánchez E. (2023). Polyphenols: Natural preservatives with promising applications in food, cosmetics and pharma industries; problems and toxicity associated with synthetic preservatives; impact of misleading advertisements; recent trends in preservation and legislation. Materials.

[70] Fredsgaard M., Fussy A., Nybo G.K., Papenbrock J., Hulkko L.S.S., Dadjoo M., Chaturvedi T., Thomsen M.H. (2026). Polyphenols in food and food wastes: Extraction, isolation, and health applications. Food Chem. Mol. Sci..

[71] Shi L., Zhao W., Yang Z., Subbiah V., Suleria H.A.R. (2022). Extraction and characterization of phenolic compounds and their potential antioxidant activities. Environ. Sci. Pollut. Res..

[72] Ayub H., Ahmad H., Zehra S.H., Ramzan K., Arif M.A., Tariq N., Capucchio M.T., Mugabi R., Sharma A., Nayik G.A. (2026). Anthocyanins from fruit and vegetable waste: Biosynthesis, extraction, and gut health benefits. Food Chem. X.

[73] Tanumihardjo S., Suri D., Simon P., Goldman I., Caballero B., Finglas P., Toldrá F. (2016). Vegetables of temperate climates: Carrot, parsnip, and beetroot. Encycl. Food Health.

[74] Knez E., Kadac-Czapska K., Dmochowska-Ślęzak K., Grembecka M. (2022). Root vegetables—Composition, health effects, and contaminants. Int. J. Environ. Res. Public Health.

[75] Bas-Bellver C., Barrera C., Betoret N., Seguí L. (2020). Turning agri-food cooperative vegetable residues into functional powdered ingredients for the food industry. Sustainability.

[76] Zambela A., Dias M.C., Guilherme R., Lorenzo P. (2025). Valorization of Agri-Food Waste to Promote Sustainable Strategies in Agriculture and Improve Crop Quality with Emphasis on Legume Crop Residues. Agronomy.

[77] Chauhan A., Islam F., Imran A., Ikram A., Zahoor T., Khurshid S., Shah M.A. (2023). A review on waste valorization, biotechnological utilization, and management of potato. Food Sci. Nutr..

[78] Ding H., Liu M. (2024). From root to seed: Unearthing the potential of carrot processing and comprehensive utilization. Food Sci. Nutr..

[79] Maranón E., Sastre H. (1991). Ion exchange equilibria of heavy metals onto chemically modified apple residues. Solvent Extr. Ion Exch..

[80] Zhou Y., Tian Y., Yang B. (2023). Root vegetable side streams as sources of functional ingredients for food, nutraceutical and pharmaceutical applications: The current status and future prospects. Trends Food Sci. Technol..

[81] Bharathi S.D., Baldia A., Aktas E., Roy D.D., Dubey K.K., Kumar G.V., Kumar V., Kundu D., Jacob S. (2025). Systematic valorisation and circular bioeconomy prospects from potato wastes: A review. Bioresour. Technol. Rep..

[82] Khanal S., Karimi K., Majumdar S., Kumar V., Verma R., Bhatia S.K., Kuca K., Esteban J., Kumar D. (2024). Sustainable utilization and valorization of potato waste: State of the art, challenges, and perspectives. Biomass Convers. Biorefinery.

[83] Wu D. (2016). Recycle technology for waste residue in potato starch processing: A review. Procedia Environ. Sci..

[84] Almeida P.V., Castro L.M., Klepacz-Smółka A., Gando-Ferreira L.M., Quina M.J. (2025). Leveraging Potato Chip Industry Residues: Bioenergy Production and Greenhouse Gas Mitigation. Sustainability.

[85] Javed A., Ahmad A., Tahir A., Shabbir U., Nouman M., Hameed A. (2019). Potato peel waste-its nutraceutical, industrial and biotechnological applacations. AIMS Agric. Food.

[86] Perez-Chabela M.D.L., Cebollón-Juárez A., Bosquez-Molina E., Totosaus A. (2022). Mango peel flour and potato peel flour as bioactive ingredients in the formulation of functional yogurt. Food Sci. Technol..

[87] Yuan M., Huan X., Yang X., Fan M., Yin J., Ma Y., Deng B., Cao H., Han Y., Xu F. (2024). Simultaneous extraction of five heavy metal ions from root vegetables via dual-frequency ultrasound-assisted enzymatic digestion. Food Chem..

[88] Asrade B., Ketema G. (2023). Determination of the selected heavy metal content and its associated health risks in selected vegetables marketed in Bahir Dar town, Northwest Ethiopia. J. Food Qual..

[89] Fonge B.A., Larissa M.T., Egbe A.M., Afanga Y.A., Fru N.G., Ngole-Jeme V.M. (2021). An assessment of heavy metal exposure risk associated with consumption of cabbage and carrot grown in a tropical Savannah region. Sustain. Environ..

[90] Muthurajan M., Veeramani A., Rahul T., Gupta R.K., Anukiruthika T., Moses J., Anandharamakrishnan C. (2021). Valorization of food industry waste streams using 3D food printing: A study on noodles prepared from potato peel waste. Food Bioprocess Technol..

[91] Ben Taher I., Fickers P., Chniti S., Hassouna M. (2017). Optimization of enzymatic hydrolysis and fermentation conditions for improved bioethanol production from potato peel residues. Biotechnol. Prog..

[92] Fradinho P., Oliveira A., Domínguez H., Torres M., Sousa I., Raymundo A. (2020). Improving the nutritional performance of gluten-free pasta with potato peel autohydrolysis extract. Innov. Food Sci. Emerg. Technol..

[93] Kaur G.J., Kumar D., Orsat V., Singh A. (2022). Assessment of carrot rejects and wastes for food product development and as a biofuel. Biomass Convers. Biorefinery.

[94] Kohli D., Champawat P., Mudgal V. (2025). Evaluation of Post-Harvest Peeling Treatments on Peeling Ease, Peeling performance and Biochemical Composition of Asparagus Roots (*Asparagus racemosus* L.). J. Appl. Res. Med. Aromat. Plants.

[95] Vescovo D., Manetti C., Ruggieri R., Spizzirri U.G., Aiello F., Martuscelli M., Restuccia D. (2025). The valorization of potato peels as a functional ingredient in the food industry: A comprehensive review. Foods.

[96] Gebrechristos H.Y., Chen W. (2018). Utilization of potato peel as eco-friendly products: A review. Food Sci. Nutr..

[97] Das S.R., Basak N. (2025). Enhancing biohydrogen production by optimization of waste potato concentration in dark and photo fermentation. J. Clean. Prod..

[98] Jayesree N., Hang P.K., Priyangaa A., Krishnamurthy N.P., Ramanan R.N., Turki M.A., Charis M.G., Ooi C.W. (2021). Valorisation of carrot peel waste by water-induced hydrocolloidal complexation for extraction of carotene and pectin. Chemosphere.

[99] Puligundla P., Mok C. (2021). Valorization of sugar beet pulp through biotechnological approaches: Recent developments. Biotechnol. Lett..

[100] Šeremet D., Durgo K., Jokić S., Huđek A., Vojvodić Cebin A., Mandura A., Jurasović J., Komes D. (2020). Valorization of banana and red beetroot peels: Determination of basic macrocomponent composition, application of novel extraction methodology and assessment of biological activity in vitro. Sustainability.

[101] Şenol H., Açıkel Ü., Oda V. (2021). Anaerobic digestion of sugar beet pulp after acid thermal and alkali thermal pretreatments. Biomass Convers. Biorefinery.

[102] El-Beltagi H.S., El-Mogy M.M., Parmar A., Mansour A.T., Shalaby T.A., Ali M.R. (2022). Phytochemical characterization and utilization of dried red beetroot (*Beta vulgaris*) peel extract in maintaining the quality of Nile Tilapia fish fillet. Antioxidants.

[103] Ma X., Duan D., Ji Y., Du Z., Shi L., Chen X. (2025). Structural analysis and flocculation performance of ASP-1: A polysaccharide bioflocculant from asparagus root waste. Ind. Crops Prod..

[104] Ibrahim R., Fayez S., Eltanany B., Abu-Elghait M., Demerdash A., Badawy M., Pont L., Benavente F., Saber F. (2024). Agro-byproduct valorization of radish and turnip leaves and roots as new sources of antibacterial and antivirulence agents through metabolomics and molecular networking. Sci. Hortic..

[105] Francavilla M., Marone M., Marasco P., Contillo F., Monteleone M. (2021). Artichoke Biorefinery: From Food to Advanced Technological Applications. Foods.

[106] Twarogowska A., Van Poucke C., Van Droogenbroeck B. (2020). Upcycling of Belgian endive (Cichorium intybus var. foliosum) by-products. Chemical composition and functional properties of dietary fibre root powders. Food Chem..

[107] Yu S., Chu Y., Tung Y., Su Z. (2025). Thermal Ageing of Black Garlic Enhances Cellular Antioxidant Potential Through the Activation of the Nrf2-Mediated Pathway. Food Technol. Biotechnol..

[108] Qiu Z., Zheng Z., Xiao H. (2025). Sustainable valorization of garlic byproducts: From waste to resource in the pursuit of carbon neutrality. Compr. Rev. Food Sci. Food Saf..

[109] Tegoundio D., Nguikwie S., Boum A. (2025). Modeling and optimization of turmerone concentrations from fermented turmeric waste essential oil via ANN and statistical techniques. Ind. Crops Prod..

[110] Trak D., Kabak B., Arslan Y., Kendüzler E. (2025). Evaluation of adsorption performance of CuO and ZnO nanoparticles synthesized by green method for treatment of Cr(VI)-polluted waters. J. Ind. Eng. Chem..

[111] Shambu B., Rajeshwari K., Bindya S., Hemavathi A., Keerthi M., Lakshmi S., Singh P., Shivamallu C., Abass K., Stupin V. (2026). Turmeric essential oil infused pectin blended sodium alginate polymer as sustainable food packaging material. Sci. Rep..

[112] Kwon H., Lim D.J., Choi C. (2025). Prevention of foodborne viruses and pathogens in fresh produce and root vegetables. Adv. Food Nutr. Res..

[113] Huang J., Hu Z., Li G., Hu L., Chen J., Hu Y. (2022). Make your packaging colorful and multifunctional: The molecular interaction and properties characterization of natural colorant-based films and their applications in food industry. Trends Food Sci. Technol..

[114] Bao Y., Cui H., Si X., Li J., Huang T., Li B. (2025). Anthocyanin-based indicator labels for intelligent food packaging: Mechanisms, multiscale regulation, and future perspectives for enhancing color-response performance. Trends Food Sci. Technol..

[115] Lakshmikanthan M., Muthu S., Krishnan K., Altemimi A.B., Haider N.N., Govindan L., Selvakumari J., Alkanan Z.T., Cacciola F., Francis Y.M. (2024). A comprehensive review on anthocyanin-rich foods: Insights into extraction, medicinal potential, and sustainable applications. J. Agric. Food Res..

[116] Khoo H.E., Azlan A., Tang S.T., Lim S.M. (2017). Anthocyanidins and anthocyanins: Colored pigments as food, pharmaceutical ingredients, and the potential health benefits. Food Nutr. Res..

[117] Ghareaghajlou N., Hallaj-Nezhadi S., Ghasempour Z. (2021). Red cabbage anthocyanins: Stability, extraction, biological activities and applications in food systems. Food Chem..

[118] Corrêa R.C.G., Garcia J.A.A., Correa V.G., Vieira T.F., Bracht A., Peralta R.M. (2019). Pigments and vitamins from plants as functional ingredients: Current trends and perspectives. Adv. Food Nutr. Res..

[119] Hernández-Ruiz R.G., Olivares-Ochoa X.C., Salinas-Varela Y., Guajardo-Espinoza D., Roldán-Flores L.G., Rivera-Leon E.A., López-Quintero A. (2025). Phenolic compounds and anthocyanins in legumes and their impact on inflammation, oxidative stress, and metabolism: Comprehensive review. Molecules.

[120] Bender L.E., Gayger A.L., Berwian G.F., Colla L.M., Chiomento J.L.T. (2026). Fruit Waste as a Resource for Biofuel Production and High-Value-Added Compounds. Processes.

[121] Núñez-Pérez J., Burbano-García J.L., Espín-Valladares R., Lara-Fiallos M.V., DelaVega-Quintero J.C., Cevallos-Vallejos M.A., Pais-Chanfrau J.-M. (2026). Cascade Valorisation of Lemon-Processing Residues (Part I): Current Trends in Green Extraction Technologies and High-Value Bioactive Recovery. Foods.

[122] Wani F.A., Rashid R., Jabeen A., Brochier B., Yadav S., Aijaz T., Makroo H., Dar B. (2021). Valorisation of food wastes to produce natural pigments using non-thermal novel extraction methods: A review. Int. J. Food Sci. Technol..

[123] Pal P., Singh A.K., Srivastava R.K., Rathore S.S., Sahoo U.K., Subudhi S., Sarangi P.K., Prus P. (2024). Circular bioeconomy in action: Transforming food wastes into renewable food resources. Foods.

[124] Clifford T., Howatson G., West D.J., Stevenson E.J. (2015). The potential benefits of red beetroot supplementation in health and disease. Nutrients.

[125] Punia Bangar S., Singh A., Chaudhary V., Sharma N., Lorenzo J.M. (2023). Beetroot as a novel ingredient for its versatile food applications. Crit. Rev. Food Sci. Nutr..

[126] Duval A.M. (2020). Valorization of Carrot Processing Waste. Master’s Thesis.

[127] Patras A. (2019). Stability and colour evaluation of red cabbage waste hydroethanolic extract in presence of different food additives or ingredients. Food Chem..

[128] Langsdorf A., Volkmar M., Holtmann D., Ulber R. (2021). Material utilization of green waste: A review on potential valorization methods. Bioresour. Bioprocess..

[129] Tenorio A.T., Gieteling J., De Jong G.A., Boom R.M., Van Der Goot A.J. (2016). Recovery of protein from green leaves: Overview of crucial steps for utilisation. Food Chem..

[130] Thivya P., Reddy N.B.P., Yuvraj K.B., Sinija V.R. (2023). Recent advances and perspectives for effective utilization of onion solid waste in food packaging: A critical review. Rev. Environ. Sci. Bio-Technol..

[131] Zhao S., Xiong F., Li J., Ye Z., Wang L., Wang T., Zhou G. (2024). Metabolomic characteristics and anthraquinones accumulation patterns of Rhubarb in different tissues and roots from different developmental stages. Food Biosci..

[132] Kim D., Iida F., Joo N. (2022). Texture properties of parsnip (*Pastinaca sativa* L.) for the elderly base on the enzyme treatment. Int. J. Food Sci. Technol..

[133] Sanchez-Salvador J., Marques M., Brito M., Negro C., Monte M., Manrique Y., Santos R., Blanco A. (2022). Valorization of Vegetable Waste from Leek, Lettuce, and Artichoke to Produce Highly Concentrated Lignocellulose Micro- and Nanofibril Suspensions. Nanomaterials.

[134] Shen Y., Jarboe L., Brown R., Wen Z. (2015). A thermochemical–biochemical hybrid processing of lignocellulosic biomass for producing fuels and chemicals. Biotechnol. Adv..

[135] Voigt C.A. (2020). Synthetic biology 2020–2030: Six commercially-available products that are changing our world. Nat. Commun..

[136] De D., Sai M.S.N., Aniya V., Satyavathi B. (2021). Strategic biorefinery platform for green valorization of agro-industrial residues: A sustainable approach towards biodegradable plastics. J. Clean. Prod..

[137] Elliott D.C., Biller P., Ross A.B., Schmidt A.J., Jones S.B. (2015). Hydrothermal liquefaction of biomass: Developments from batch to continuous process. Bioresour. Technol..

[138] Gollakota A.R.K., Kishore N., Gu S. (2018). A review on hydrothermal liquefaction of biomass. Renew. Sustain. Energy Rev..

[139] Sriariyanun M., Gundupalli M.P., Phakeenuya V., Phusamtisampan T., Cheng Y.-S., Venkatachalam P. (2023). Biorefinery approaches for production of cellulosic ethanol fuel using recombinant engineered microorganisms. J. Appl. Sci. Eng.

[140] Areeya S., Panakkal E.J., Kunmanee P., Tawai A., Amornraksa S., Sriariyanun M., Kaoloun A., Hartini N., Cheng Y.-S., Kchaou M. (2024). A review of sugarcane biorefinery: From waste to value-added products. Appl. Sci. Eng. Prog..

[141] Ahmad Z., Rauf A., Orhan I.E., Mubarak M.S., Akram Z., Islam M.R., Imran M., Edis Z., Kondapavuluri B.K., Thangavelu L. (2025). Antioxidant Potential of Polyphenolic Compounds, Sources, Extraction, Purification and Characterization Techniques: A Focused Review. Food Sci. Nutr..

[142] Li B.-Y., Xia Z.-Y., Gou M., Sun Z.-Y., Huang Y.-L., Jiao S.-B., Dai W.-Y., Tang Y.-Q. (2022). Production of volatile fatty acid from fruit waste by anaerobic digestion at high organic loading rates: Performance and microbial community characteristics. Bioresour. Technol..

[143] Lateef A., Darwesh O.M., Matter I.A. (2021). Microbial nanobiotechnology: The melting pot of microbiology, microbial technology and nanotechnology. Microbial Nanobiotechnology: Principles and Applications.

[144] Rocha-Meneses L., Hari A., Inayat A., Shanableh A., Abdallah M., Ghenai C., Shanmugam S., Kikas T. (2022). Application of nanomaterials in anaerobic digestion processes: A new strategy towards sustainable methane production. Biochem. Eng. J..

[145] Chaudhary R., Kumar S., Sharma V. (2025). Agricultural biowaste valorization towards circular economy: Nanoparticles synthesis and biopolymers extraction for wastewater remediation. Bioresour. Technol. Rep..

[146] Coman V., Teleky B.-E., Mitrea L., Martău G.A., Szabo K., Călinoiu L.-F., Vodnar D.C. (2020). Bioactive potential of fruit and vegetable wastes. Adv. Food Nutr. Res..

[147] Carrot CarbonCloud Benchmark. https://apps.carboncloud.com/climatehub/product-reports/id/89115431439.

[148] Sato N., Shimizu N. (2025). Subcritical solvent extraction and microencapsulation of flavonoids from onion skin waste. Appl. Food Res..

[149] Benito-Román Ó., Alonso-Riaño P., De Cerio E.D., Sanz M., Beltrán S. (2022). Semi-continuous hydrolysis of onion skin wastes with subcritical water: Pectin recovery and oligomers identification. J. Environ. Chem. Eng..

[150] Mathialagan R., Mansor N., Shamsuddin M.R., Uemura Y., Majeed Z. (2017). Optimisation of ultrasonic-assisted extraction (UAE) of allicin from garlic (*Allium sativum* L.). Chem. Eng. Trans..

[151] Shen G., Li B., Xu Y., Chen X., Katiyar S., Zhu L., Xie L., Han Q., Qiu X., Wu X. (2024). Waste biomass garlic stem-derived porous carbon materials as high-capacity and long-cycling anode for lithium/sodium-ion batteries. J. Colloid Interface Sci..

[152] Vo T.P., Nguyen N.H., Pham B.G., La N.A.T., Pham X.D.A., Nguyen D.Q., Nguyen H.N., Pham G.B. (2025). Application of ultrasound-microwave-assisted extraction to extract phenolic and terpenoid compounds from celery stalk. J. Agric. Food Res..

[153] Kutlu N. (2025). The impact of osmotic dehydration and microwave drying process conditions on the quality characteristics of celery roots (Apium graveolens l. Subsp. rapaceum). Therm. Sci. Eng. Prog..

[154] Carboncloud Benchmark Beetroot, Fresh. https://apps.carboncloud.com/climatehub/product-reports/id/151627332574.

[155] Cakmak H., Dekker M. (2022). Optimization of cellulosic fiber extraction from parsley stalks and utilization as filler in composite biobased films. Foods.

[156] CarbonCloud Potatoes. https://apps.carboncloud.com/climatehub/product-reports/id/89722963982.

[157] HUMA Bio Energizer® Cuts Sludge Hauling Costs for Potato Wastewater Treatment Plant. https://huma.us/environmental/blog/bio-energizer-cuts-sludge-hauling-costs-for-potato-wastewater-treatment-plant/.

[158] Geng X., Gong Z., Tian W., Zhuang M., Shang H., Chen Y., Li J., Lv Y., Bai K. (2025). Nutritional and phytochemical characterization of radish leaves: A comprehensive overview. Foods.

[159] Horseradish Powder, Europe. https://apps.carboncloud.com/climatehub/product-reports/id/2163615238005.

[160] Carrots and Turnips. https://apps.carboncloud.com/climatehub/agricultural-reports/benchmarks/a6b461b5-e2d9-4a84-a077-e84a110ab409#:~:text=Carrots%20and%20turnips%2C%20World%20%C2%B7%200.16,CO%E2%82%82e/kg%20%7C%20Verified%20by%20CarbonCloud.

[161] Artichoke Hearts. https://apps.carboncloud.com/climatehub/product-reports/id/978223320421.

[162] Carlos R., Marina M., Garcia M. (2025). Recovery of proteins and bioactive compounds from turmeric (*Curcuma longa*) and ginger (*Zingibber officinale*) wastes using sustainable extraction techniques. Tentative identification of main extracted compounds by UHPLC-Q-TOF-MS/MS. Adv. Sample Prep..

[163] Maric L., Malesic E., Tusek A., Benkovic M., Valinger D., Jurina T., Kljusuric J. (2020). Effects of drying on physical and chemical properties of root vegetables: Artificial neural network modelling. Food Bioprod. Process..

[164] Castro L.E.N., Sganzerla W.G., Silva A.P.G., John O.D., Barroso T.L.C.T., Rostagno M.A., Forster-Carneiro T. (2025). Sustainable extraction methods for the recovery of polyphenolic compounds from grape pomace and its biological properties: A comprehensive review. Phytochem. Rev..

[165] Ariyanta H.A., Sholeha N.A., Fatriasari W. (2025). Current and future outlook of research on renewable cosmetics derived from biomass. Chem. Biodivers..

[166] Paini J., Benedetti V., Ail S.S., Castaldi M.J., Baratieri M., Patuzzi F. (2022). Valorization of wastes from the food production industry: A review towards an integrated agri-food processing biorefinery. Waste Biomass Valorization.

[167] Capanoglu E., Nemli E., Tomas-Barberan F. (2022). Novel approaches in the valorization of agricultural wastes and their applications. J. Agric. Food Chem..

[168] Zabed H., Sahu J., Suely A., Boyce A., Faruq G. (2017). Bioethanol production from renewable sources: Current perspectives and technological progress. Renew. Sustain. Energy Rev..

[169] Broda M., Yelle D.J., Serwańska K. (2022). Bioethanol production from lignocellulosic biomass—Challenges and solutions. Molecules.

[170] Huzir N.M., Asmadi A.A., Rosly M.B., Tamunaidu P., Amin A.N.R. (2026). Composting as a pathway for organic waste valorization: Substrate performance, process strategies, and quality benchmarks. J. Mater. Cycles Waste Manag..

[171] Villanueva-Galindo E., Vital-Jácome M., Moreno-Andrade I. (2023). Dark fermentation for H2 production from food waste and novel strategies for its enhancement. Int. J. Hydrogen Energy.

[172] Brusselaers J., Van Der Linden A. (2020). Bio-waste in Europe—Turning challenges into opportunities. Environ. Econ..

[173] Bădan D.N., Dumitru E.A. (2020). The bioenergy potential of agricultural residues in Romania. Sci. Pap. Ser. Manag. Econ. Eng. Agric. Rural Dev..

[174] Vintilă T., Neo S. (2011). Biogas in Romanian agriculture, present and perspectives. Sci. Pap. Anim. Sci. Biotechnol..

[175] Barrios C., Lucas S., García-Cubero M., Coca M., López-Linares J. (2025). Biorefinery based on discarded red beetroot: Production of bioactive compounds and 2,3-butanediol. Biomass Convers. Biorefinery.

[176] Mirheli M., Dinani S. (2018). Extraction of β-carotene pigment from carrot processing waste using ultrasonic-shaking incubation method. J. Food Meas. Charact..

[177] Board C.S. Bio-Plastics from Potato Peels By Chip[s] Board. https://www.designnuance.com/bio-plastics-from-potato-peels-by-chips-board/.

[178] Hudek-Pietras A., Korzeniowski Ł., Lewandowski M., Wądrzyk M. (2026). Dual-stage valorization of carrot pomace waste biomass by aqueous extraction and acid hydrolysis for saccharide recovery. Food Bioprod. Process..

[179] Alherbawi M., Parthasarathy P., Elkhalifa S., Al-Ansari T., McKay G. (2024). Techno-economic and environmental analyses of the pyrolysis of food waste to produce bio-products. Heliyon.

[180] Joensuu K., Harrison E., Hartikainen H. (2022). What to Do with Food Waste? A Holistic Feasibility Framework to Evaluate Different Solutions. Sustainability.

[181] Degirmenci H., Altuntas O. (2026). Life cycle assessment of hydrochar production from sugar beet pulp: Analyzing post-drying techniques. Sustain. Energy Technol. Assess..

[182] Berglund L., Breedveld L., Oksman K. (2020). Toward eco-efficient production of natural nanofibers from industrial residue: Eco-design and quality assessment. J. Clean. Prod..

